# Specimen-specific drift of densities defines distinct subclasses of extracellular vesicles from human whole saliva

**DOI:** 10.1371/journal.pone.0249526

**Published:** 2021-04-08

**Authors:** Satoshi Yamamoto, Kohji Okamura, Risa Fujii, Takamasa Kawano, Koji Ueda, Yasutomo Yajima, Kiyotaka Shiba

**Affiliations:** 1 Division of Protein Engineering, Cancer Institute, Japanese Foundation for Cancer Research, Tokyo, Japan; 2 Department of Oral and Maxillofacial Implantology, Tokyo Dental College, Tokyo, Japan; 3 Department of Systems BioMedicine, National Center for Child Health and Development, Tokyo, Japan; 4 Cancer Precision Medicine Center, Japanese Foundation for Cancer Research, Tokyo, Japan; 5 Department of Oral Oncology, Oral and Maxillofacial Surgery, Tokyo Dental College, Chiba, Japan; J. Heyrovsky Institute of Physical Chemistry, CZECH REPUBLIC

## Abstract

Extracellular vesicles (EVs) in body fluids constitute heterogenous populations, which mirror their diverse parental cells as well as distinct EV-generation pathways. Various methodologies have been proposed to differentiate EVs in order to deepen the current understanding of EV biology. Equilibrium density-gradient centrifugation has often been used to separate EVs based on their buoyant densities; however, the standard conditions used for the method do not necessarily allow all EVs to move to their equilibrium density positions, which complicates the categorization of EVs. Here, by prolonging ultracentrifugation time to 96 h and fractionating EVs both by floating up or spinning down directions, we allowed 111 EV-associated protein markers from the whole saliva of three healthy volunteers to attain equilibrium. Interestingly, the determined buoyant densities of the markers drifted in a specimen-specific manner, and drift patterns differentiated EVs into at least two subclasses. One class carried classical exosomal markers, such as CD63 and CD81, and the other was characterized by the molecules involved in membrane remodeling or vesicle trafficking. Distinct patterns of density drift may represent the differences in generation pathways of EVs.

## Introduction

Body fluids contain large numbers of extracellular vesicles (EVs), which collectively refer to the membranous particles secreted from cells [1]. These EVs are released from various types of parental cells via distinct generation pathways, making EVs heterogeneous populations [1–4]. Numerous studies have revealed the pivotal roles of EVs in various biological activities, including normal development such as spermatogenesis [5] as well as the progression of various disorders, including cancer, neurodegenerating diseases, etc. [6–8]; however, a complete picture of EVs has not yet been well elucidated, and an in-depth understanding of these EV-involved biological activities requires systemized classification of the heterogeneous EV populations [1, 9]. When certain molecules (proteins, nucleic acids, lipids, or glycans) are specifically linked to a parental cell or a certain generation route, they can be used as molecular tags to concentrate or isolate the EV subclass [10–12]. In most cases, however, the expressions of EV markers overlap between cell types or generation routes and, therefore, the strategy does not always work for defining specific EV subclasses [1, 2]. Besides the marker-based classifications, the physical-chemical properties of EVs have also been used to define subclasses of EVs. In particular, sedimentation profile of EVs in centrifugation have been frequently used to fractionate EVs [13–15]. For instance, a sequential centrifugation at 300, 2,000, 10,000, and 160,000 g centrifugation deposited cells, large EVs, medium EVs, and small EVs as pellets, respectively. In this differential centrifugation experiment, the pelleted fraction from 160,000 g (P160) is often called "exosomes", although the fraction contains nonvesicular entities as well as nonendosome-originated EVs [4, 15–18]. In some instances, the P160 fraction is further differentiated based on their densities running through the density-gradient medium [2, 15, 17, 19, 20]. This method is called "equilibrium density-gradient centrifugation," in which molecules are expected to stop moving at a point where the density of the particles equals the density of the solution around them [14]. However, the standard conditions used for the method do not necessarily allow all EVs to move to a state of equilibrium; that is, some EVs still remain in the process of movement through density-gradient media, complicating the categorization of EVs based on densities [2, 20–22]. For this reason, to determine the innate densities of subclasses of EVs, we first prolonged the ultracentrifugation time from 17 h to 96 h and allowed the EVs to move in two directions: floating up and spinning down. With this protocol, 111 proteins out of 1,420 identified from mass-spectroscopy achieved equilibrium from the whole saliva sample of three healthy volunteers, because they resided at the identical density fraction irrespective of movement directions. Interestingly, the determined buoyant densities of the markers drifted in a specimen-specific manner, and the pattern of these drifts differentiate 111 markers into two subclasses. One class contained classical exosomal markers, and the other carried molecules involved in membrane remodeling or vesicle trafficking. Distinct patterns of density drift may represent differences in the generation pathways of the EVs. Our findings would boost the development of a salivary-based liquid biopsy system for oral-associated lesions as well as for systemic diseases [23–25].

## Material and methods

### Reagents

Iodixanol (OptiPrep™), 4-(2-hydroxyethyl)-1-piperazine ethanesulfonic acid (HEPES) and polyoxyethylene (20) sorbitan monolaurate (Tween-20) (P1379) were purchased from Axis-Shield PoC (1114542, Oslo, Norway), Nacalai Tesque, Inc. (17547–95, Kyoto, Japan) and Sigma-Aldrich (MO, USA), respectively.

### Whole saliva collection

Whole saliva or oral fluid (OF) samples were collected following the modified method by Iwai et al. [20], after approval by the medical ethics committee at the Japanese Foundation for Cancer Research (approval number: JFCR 2013–1112 and 2016–1097). This study was conducted according to the principles of the Declaration of Helsinki, and the informed consent was obtained from all participants. Briefly, 30 mL of whole OFs were collected from three healthy volunteers with a mean age of 30 years (28, 30, and 34 years), who were prohibited from eating, drinking, smoking, and performing oral hygiene procedures for at least 1h before sampling. Immediately after collection, specimens were divided between 2 x 15 mL in 50 mL plastic tubes (227261, Greiner Bio-One International, Kremsmünster, Austria), mixed with 2 x 15 mL of phosphate buffer saline (PBS, 137 mM NaCl, 2.68 mM KCl, 8.10 mM Na_2_HPO_4_, 1.47 mM KH_2_PO_4_, pH 7.4) and were sonicated using a closed-type sonication system (Bioruptor, Diagenode, Inc., Liege, Belgium) at a total run time of 10 min, comprised of 30-sec pulses, 1-min intervals at medium power with level of three to reduce the viscosity of OF samples. The necessity of the sonication step for density-gradient-based isolation and its effects on EVs are shown in S1 Fig.

### Extensive equilibrium density-gradient centrifugation

After sonication, specimens were centrifuged at 2,600 g for 30 min at 4°C (model 5500, Kubota, Osaka, Japan). The supernatants were collected in 38.5 mL ultraclear tubes (#344058, Beckman Coulter, CA, USA) and were centrifuged at 160,000 g for 70 min at 4°C (L-90K with SW32Ti rotor, Beckman Coulter) to prepare crude EVs. In this study, centrifugations with medium speed were omitted to analyze not only small EVs but also medium and large EVs. For downward separation, the EV pellets were resuspended in 500 μL of PBS and layered on the 8% to 47% continuous density of iodixanol in 0.02 M HEPES [4-(2-hydroxyethyl)-1-piperazine ethanesulfonic acid]/NaOH, pH 7.2, which was formed in 32 mL thickwall polycarbonate tube (#355631, Beckman Coulter) using a gradient mixer No.3 (SAN4024, Sanplatec, Osaka, Japan). For upward separation, the pellets were resuspended in 500 μL of 47% iodixanol in 0.02 M HEPES/NaOH, pH 7.2 and put at the bottom of the centrifugation tube. Subsequently, continuous density gradient was formed as described above. After centrifugation (L-90K with SW32Ti rotor) at 160,000 g for 96 h or 17 h at 4°C, 10 individual 3-mL fractions were collected in ultraclear tubes (#344058, Beckman Coulter) from the top, and the density of each fraction was measured using a refractometer (RX-5000a, Atago Co. Ltd, Tokyo, Japan). Each fraction was centrifuged at 160,000 g for 120 min at 4°C (L-90K with SW32Ti rotor) after adding 30 mL of PBS, and pellets were resuspended in 500 μL of PBS. These samples were stored at 4°C until analysis.

### Western blotting and silver staining

For Western blotting (WB) and silver staining, 18 μL samples of each fraction were mixed with 6 μL of 4 x reducing SDS sample buffer (0.25 M Tris HCl, pH 6.8, 20% sucrose, 8% SDS, 20% 2-mercaptoethanol, 0.008% bromophenol blue) or 4x nonreducing SDS sample buffer (without 2-mercaptoethanol, for anti-CD63 antibody) followed by incubation at 95°C for 5 min. Proteins were electrophoresed through 10% or 15% polyacrylamide gel (Extra PAGE One Precast Gel, Nacalai Tesque, Inc.), and they were transferred unto PVDF membrane using the dry blotting system (iBlot, Thermo Fisher Scientific, Carlsbad, CA, USA). The blotted membrane was blocked with Blocking One (03953–95, Nacalai Tesque) for 1 h and incubated with mouse anti-CD63 (ab8219, Abcam, Cambridge, UK, 1:1,000 dilution) for 15 h at 4°C with mild shaking. After washing with TBS-T (10 mM Tris-HCl, 150mM NaCl, 0.02% Tween-20) 3 times for 5 min, the membrane was incubated with goat anti-mouse IgG (H + L)-HRP conjugate (1706516, Bio-Rad, Hercules, CA, USA, 1:2,000) for 2 h at 4°C with mild shaking, followed by 3 x 5 min washing with TBS-T and incubation with enhanced chemiluminescence (ECL) system (A-8511, C-9008, Sigma-Aldrich) The signals were imaged using ChemiDoc camera system (1708265, Bio-Rad) with default parameters. For silver staining, gels were stained using Sil-Best Stain One (Nacalai Tesque, Inc.) according to the manufacturer’s protocol. Molecular weights markers of BLUE Star Prestained Protein-Ladder (Nippon Genetics Co., Ltd., Tokyo, Japan) and DAIICHI-Ⅲ (DAIICHI PURE CHEMICALS CO., Ltd, Tokyo, Japan) were used for WB and silver staining, respectively.

### Mass spectrometric analysis

Protein samples were purified and concentrated using the 2-D Clean-Up Kit (80648451, GE Healthcare, Chicago, IL) according to the manufacturer’s instructions. Samples were then reduced in 1 × Laemmli’s sample buffer (32.9mM Tris HCl, pH6.8, 1.05% SDS, 13.15% glycerol, 0.005% bromophenol blue) with 10 mM tris(2-carboxyethyl)phosphine at 100°C for 10 min, alkylated with 50 mM iodoacetamide at ambient temperature for 45 min, and subjected to SDS-PAGE. The electrophoresis was stopped at the migration distance of 2 mm from the top edge of the separation gel. After coomassie brilliant blue-staining, protein bands were excised, destained, and cut finely prior to in-gel digestion with Trypsin/Lys-C Mix (V5071, Promega, Madison, WI, USA) at 37°C for 12 h. The resulting peptides were extracted from gel fragments and analyzed with Orbitrap Fusion Lumos mass spectrometer (Thermo Fisher Scientific, Waltham, MA) combined with UltiMate 3000 RSLC nano-flow HPLC (Thermo Fisher Scientific). The MS/MS spectra were searched against the “Homo sapiens” protein sequence database in SwissProt using Proteome Discoverer 2.2 software (Thermo Fisher Scientific), in which peptide identification filters were set at “false discovery rate < 1%”.

### Analyses of the quantified values of mass spectrometric data

Mass spectrometric data assigned with human proteins were analyzed using a custom Perl script. Each entry consisted of a specimen number, the direction of the gradient movement, fraction number, an MS quantified value, and an assigned protein ID. MS quantified values were first processed with the base 10 logarithm. Since the three saliva specimens were divided into two groups for the two opposite movements, six units of samples were available for analysis. For each protein, we searched for the fraction where the protein was the most abundantly detected within a unit. The logarithm values in a unit were normalized by the maximum. The Perl script used in these analyses is available from GitHub repository (https://github.com/yamamoto-tdc/EV-saliva).

### EV-TRACK

We have submitted all relevant data pertaining to our experiments to the EV-TRACK knowledgebase (EV-TRACK ID: EV190057).

## Results

### Extensive equilibrium density-gradient centrifugation allows more molecules to reach a state of equilibrium

**Fig 1:**
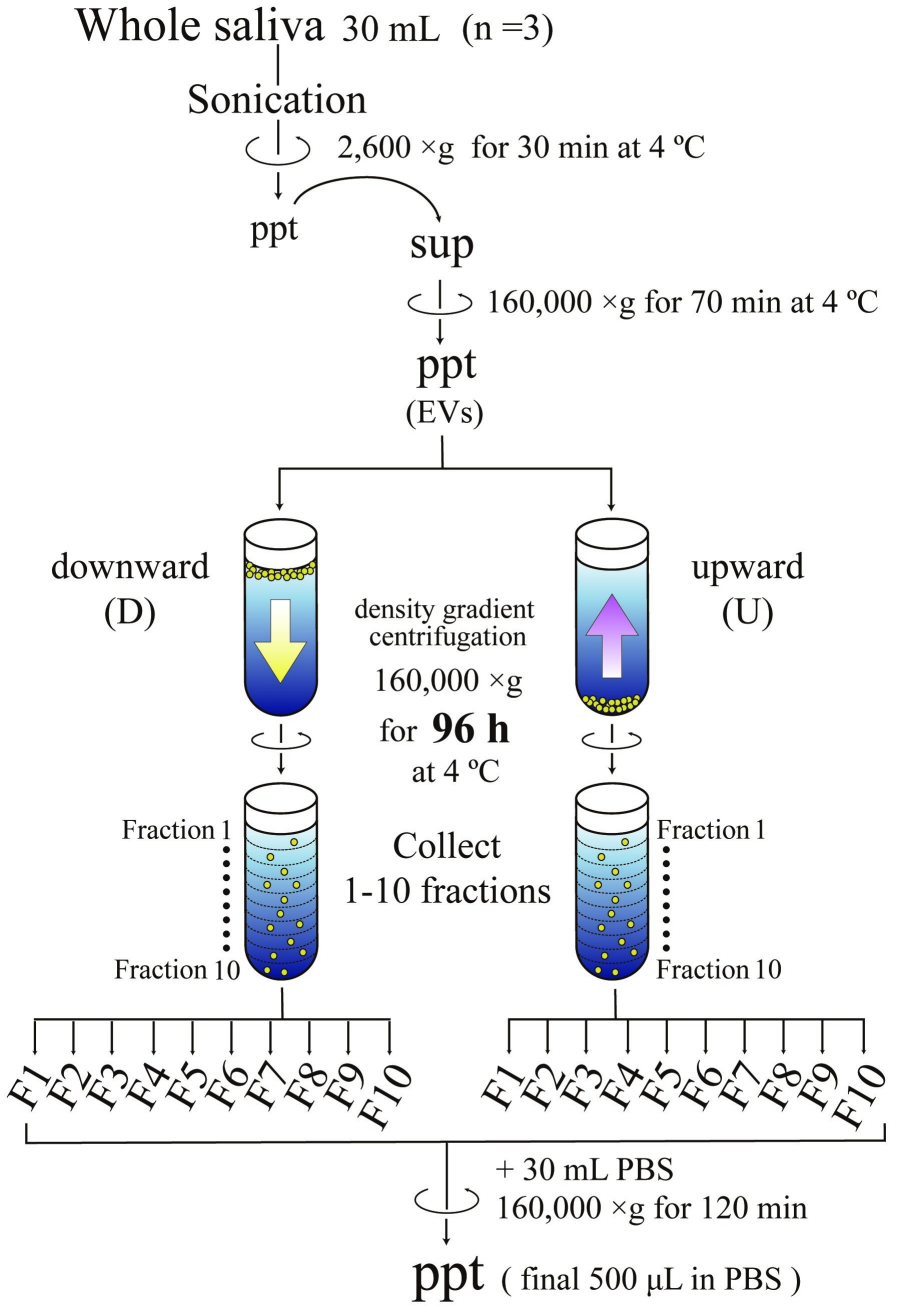
Scheme of EV fractionation using density gradient centrifugation. Large particles in whole saliva such as desquamated epithelia and blood cells were first removed by centrifugation at 2,600 g for 30 min. Then the resultant supernatants were centrifuged at 160,000 g for 70 min to obtain the pellets of particles containing various sizes of EVs. These particles were fractionated by both spinning down (downward, D) and floating up (upward, U) through the density gradient of iodixanol at 160,000 g for 96 h. After centrifugation, ten fractions (F1 to F10) were recovered from the top and analyzed after being concentrated through centrifugation at 160,000 g for 120 min.

In this study, to allow more EVs to attain their state of equilibrium in density gradient centrifugation, the running time was prolonged to 96 h from 17 h. Furthermore, the specimens that were collected from three healthy volunteers were fractionated using two methods: spinning down (downward) or floating up (upward) directions through a density gradient of iodixanol (Fig 1). After centrifugation, ten fractions (F1–F10) were collected and analyzed using SDS-PAGE and silver staining (S2 Fig). After 17 h of centrifugation, distributions of silver staining signals through fractions differed between samples centrifuged for fractionation in the downward direction and those centrifuged for fractionation in an upward direction, indicating that many of molecules were still *en route* to their corresponding density positions. In contrast, signal patterns obtained from the 96 h centrifugation were almost identical between samples centrifuged in the downward direction and those centrifuged in an upward direction, demonstrating that most molecules had already reached their state of equilibrium. To verify the states of equilibrium for specific molecules, WB analyses were conducted using anti-CD63, CD81, CD9, α-amylase, HSP70, AQP5, and TSG101 antibodies (S3 Fig). From 96 h centrifugation, all proteins except α-amylase provided the strongest signals in the identical fractions both downward and upward directions, whereas, from 17 h centrifugation, the strongest signals of CD81, HSP70, APQ5 and α-amylase were not in the identical positions between downward and upward directions, indicating that they were not in equilibrium. Although many proteins attained equilibrium in the 96 h centrifugation time, some proteins including α-amylase were still in the course of movement in the medium (S3 Fig), suggesting that these molecules themselves or their associated carriers have peculiar structures or surface properties that hinder their movement in the media.

### Idiosyncratic densities of CD63 on different specimen

**Fig 2:**
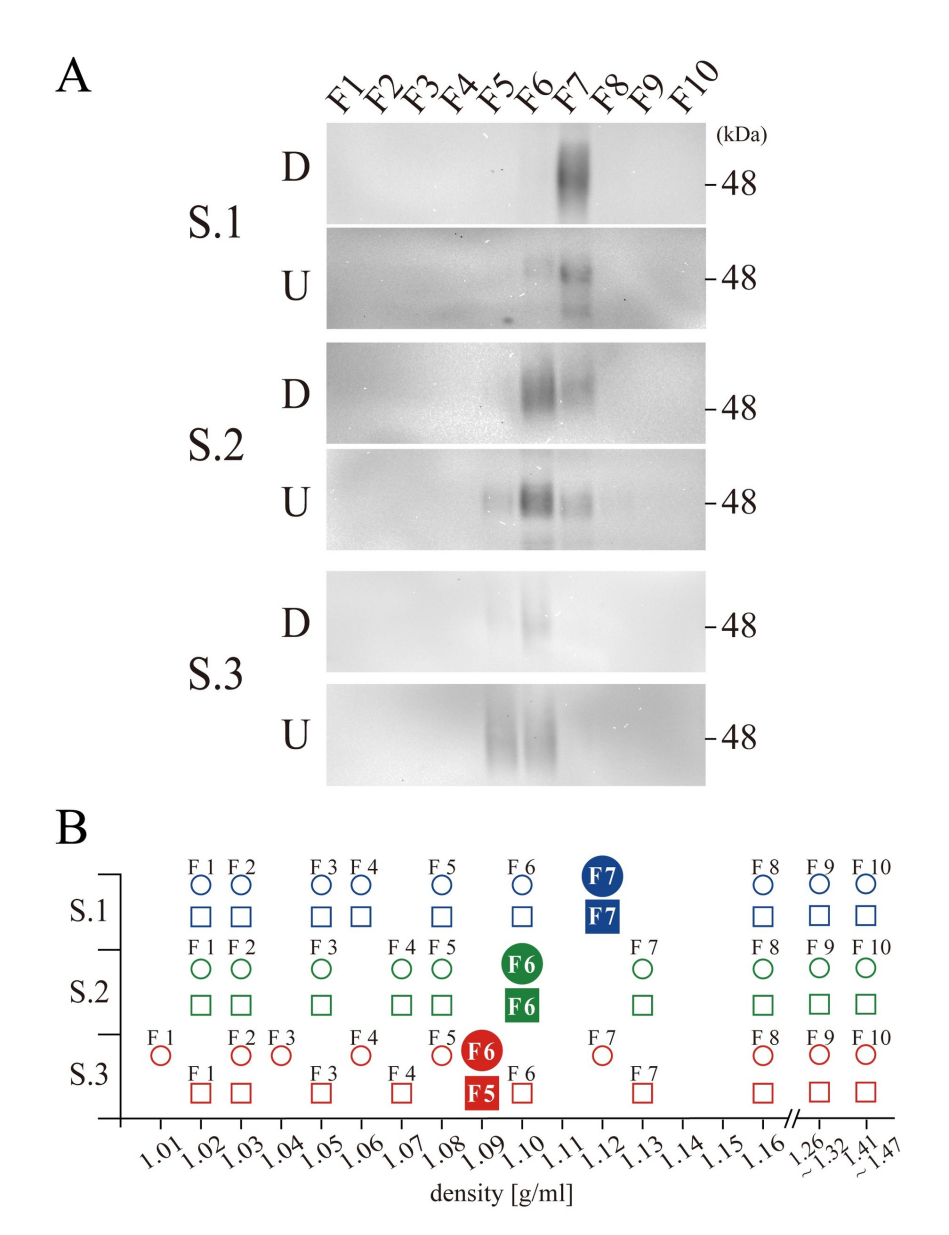
Specimen-specific density drift. (A) Western blotting for CD63 from specimen 1 (S.1), Specimen 2 (S.2) and Specimen 3 (S.3) both in the downward (D) and upward (U) centrifugation. A position of 48 KDa molecular weight marker is indicated at the right side of S.1 D panel, and the fraction numbers are indicated on the top. (B) The densities of 60 fractions from three specimens (Specimen 1 in blue, 2 in green and 3 in red) both in downward (circles) and in upward (squares) centrifugation are plotted, including those fractions with the strongest signals of CD63 (filled circles and squares).

In the preceding analyses, it should be noted that the fractions that contained CD63 had different densities among the three specimens with 96 h centrifugation. The strongest signals in WB were observed at F7 from Specimen 1 in both the downward and upward direction, and the densities of these fractions were 1.12 g/ml (Fig 2). By contrast, from Specimens 2 and 3, the fractions containing CD63 had densities of 1.10 g/ml (F6 in both the downward and upward directions) and 1.09 g/ml (F6 in downward and F5 in upward), respectively (Fig 2). Because each fraction from different tubes had slightly different density after centrifugation, a total of 60 fractions (three specimens, 10 fractions for two sample groups, one centrifuged in the downward direction and the other centrifuged in the upward directions) were mapped on the abscissa of determined densities, which clarifies specimen specific differences in densities of CD63 (Fig 2B, in which blue, green and red represent Specimen 1, 2 and 3, respectively). The density of CD63 from Specimen 2 was 1.10 g/ml. From Specimen 1, the strongest signal for CD63 was observed from the fraction with 1.12 g/ml but not from the one with 1.10 g/ml. Similarly, from Specimen 3, CD63 was localized at 1.09 g/ml but not at 1.12 g/ml. Thus, the data suggested that dominant EVs containing CD63 possessed specimen-specific idiosyncratic densities.

### Comprehensive analyses of fractionated proteins by mass spectrometry

**Fig 3:**
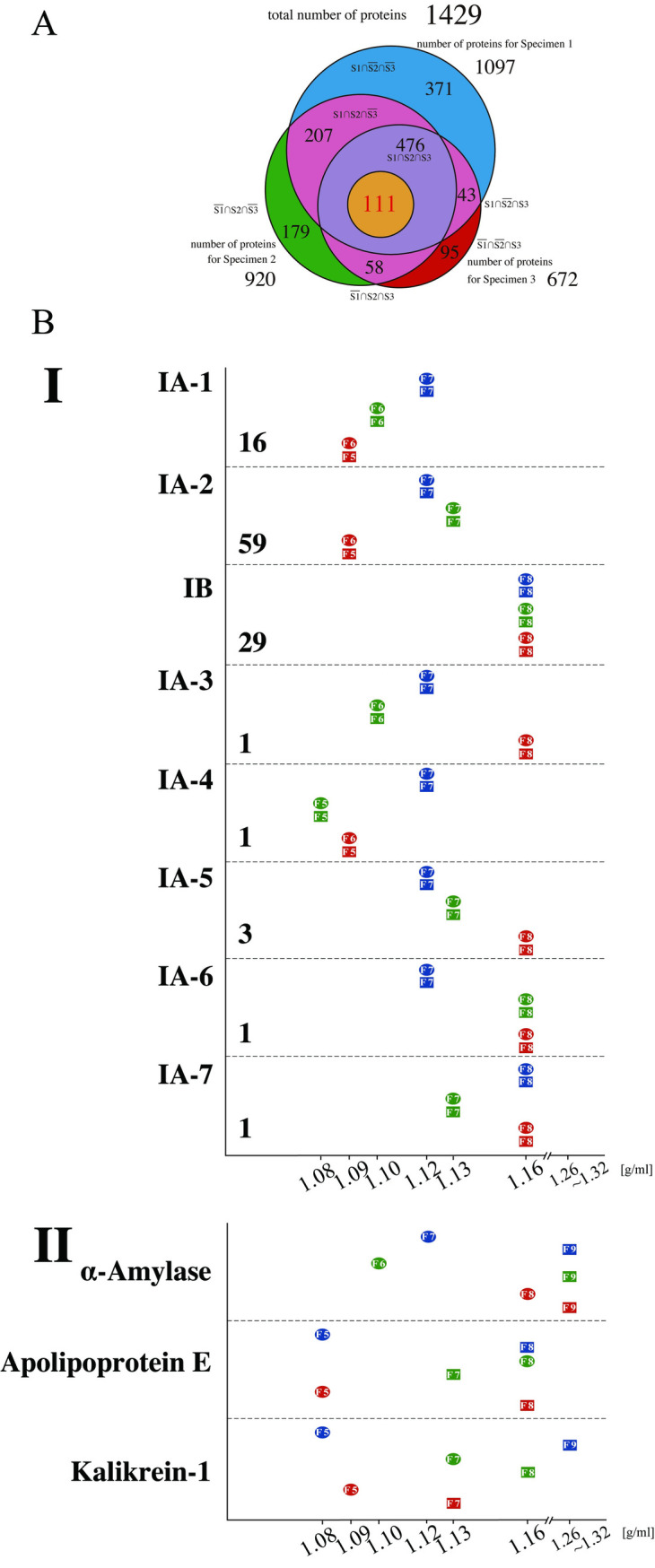
Proteomic analyses of the density fractions from three specimens in downward and upward separation. (A) Venn diagram shows the number of proteins that were detected common to Specimens 1 and 2; the number of proteins that were detected common to Specimens 1 and 3; the number of proteins that were detected common to Specimens 2 and 3; the number of proteins that were detected only in Specimen 1; the number of proteins that were detected only in Specimen 2; and the number of proteins that were detected only in Specimen 3. The blue-colored area represents the number of proteins that were detected only in Specimen 1; the green-colored area, the number of proteins that were detected only in Specimen 2; the red-colored area, the number of proteins that were detected only in Specimen 3, and the pink-colored area, the numbers of proteins that were detected common to Specimens 1 and 2, Specimens 1 and 3, and Specimens 2 and 3, respectively. The size of each circle corresponds to the number of proteins in each category. The list of proteins in each group is provided in S1 Dataset. (B) Graphs showing the densities of the fractions of each specimen in Groups IA-1 to IA-7, IB and some proteins in Group II. The horizontal axis indicates densities that were determined using the refractometer. The circles and squares indicate the fraction numbers having PFD (see text) from the downward and upward direction, respectively. The number of proteins contained in each group is indicated under the name of the group.

Western blot analyses revealed that the density of the vesicles containing CD63 differed among specimens (Fig 2). To determine if this phenomenon was specific to CD63, comprehensive analyses of the proteins of each of the 60 fractions using mass spectrometry (MS) were performed. This process identified a total of 11,749 proteins that contained 1,429 nonoverlapped proteins. The total number of proteins identified in Specimens 1, 2, and 3 were 1,097, 920 and 672, respectively. Out of them, 476 were common among all three specimens (Fig 3A). The complete list of 1,429 proteins is provided in S1 Dataset.

Among the 476 proteins that were detected from all three specimens in both sample groups (centrifuged or fractionated in the downward and upward directions), we identified those that had identical densities within a given specimen; that is, the proteins that reached a state of equilibrium. For this purpose, the total area obtained by MS was quantified with Proteome Discoverer 2.2 and used as relative quantities, which have often been employed in label‐free proteomic analyses [26]. When the density of the fraction with the highest quantitative value was the same in both the sample group centrifuged in the downward direction and the sample group centrifuged in the upward direction for a given specimen, the protein was regarded as having attained equilibrium, and this density was defined as "peak fraction density (PFD)." Out of 476 proteins, the PFD values of 111 were determined; that is, 111 proteins achieved equilibrium in all of three specimens (Fig 3A). These 111 proteins were categorized as Group I.

### Specimen-specific PFD values of 111 proteins outline two EV subpopulations

In agreement with the results of WB analyses (Fig 2), PFD values of CD63 in MS analyses were determined to be 1.12 g/ml, 1.10 g/ml, and 1.09 g/ml for Specimen 1, Specimen 2, and Specimen 3, respectively. Furthermore, out of 111 proteins, we identified 16 proteins (including CD63) that possessed an identical pattern of PFD values observed in CD63; these proteins were classified into IA-1 (Fig 3B). The remaining 95 proteins were sorted into two major groups (IA-2 and IB) and 5 minor groups (IA-3, IA-4, IA-5, IA-6 and IA-7). Subgroup IA-2 consisted of 59 proteins, and it also demonstrated idiosyncratic PFD values (1.12 g/ml, 1.13 g/ml and 1.09 g/ml for Specimens 1, 2 and 3, respectively), the pattern of which was distinct from subgroup IA-1. Unlike the proteins categorized into Group IA, 29 proteins had identical PFD values among the three specimens and were classified into IB (Fig 3B). Proteins in IB included many globulin group proteins, such as Igα1 and Igα2 (S1 Dataset). In summary, Groups IA-1 and IA-2 showed a specimen-specific drift of densities, and Group IB showed an identical high density among the specimens.

### Group IA-1 and Group IA-2 subclasses outline two EVs having distinct functionalities

Sixteen proteins classified into Group IA-1 included CD63, CD81, CD9, and others, most of which were membrane proteins or membrane anchored (*via* lipidic modifications) proteins, except for CIB1, LITAF, and UBC (Table 1). Among them, CD63, CD81, and CD9 have often been referred to as classical or canonical exosome markers.

**Table 1:** List for of proteins classified into IA-1.

Protein name	Gene	Biological function*	Expected location*	Signal peptide*	reference
***IA-1***					
Calcium and integrin-binding protein 1	CIB1	apoptotic process, cell adhesion	Cytosol	−	[27]
CD63	CD63	cell adhesion, cell migration	Integral to plasma membrane	+	[28]
CD81	CD81	cell proliferation	Integral to plasma membrane	−	[28]
CD9	CD9	cell adhesion	Integral to plasma membrane	−	[29]
Choline transporter-like protein 4	SLC44A4	acetylcholine biosynthetic process	Integral to plasma membrane	−	[29]
G-protein coupled receptor family C group 5 member C	GPRC5C	G-protein coupled receptor signaling pathway	Integral to plasma membrane	+	[29]
Glutamate carboxypeptidase 2	FOLH1 (PSMA)	unknown	Integral to plasma membrane	−	[30]
HLA class II histocompatibility antigen, DR alpha chain	HLA-DRA	immune response	Integral to plasma membrane	+	[30]
Leucine-rich repeat-containing protein 26	LRRC26	potassium channel activity	Integral to plasma membrane	+	[30]
Lipopolysaccharide-induced tumor necrosis factor-alpha factor	LITAF	endosomal protein trafficking	cytoplasmic side of plasma membrane	−	[31]
Monocyte differentiation antigen CD14	CD14	immune response	Integral to plasma membrane	+	[32]
Phospholipid scramblase 1	PLSCR1	apoptotic process	Integral to plasma membrane	−	[29]
Polyubiquitin-C	UBC	apoptotic process	Cytosol	−	[33]
Prominin-2	PROM2	pinocytotic process, endocytosis	Integral to plasma membrane	+	[34]
Protein lifeguard 3	TMBIM1	apoptotic process	Integral to plasma membrane	−	[29]
Tetraspanin-1	TSPAN1	cell migration, cell proliferation	Integral to plasma membrane	−	[29]

* Based on Uniprot (https://www.uniprot.org/).

Group IA-2 was composed of 59 proteins (Table 2), many of which are involved in membrane dynamics and membrane trafficking. Group IA-2 was also characterized by the presence of the proteins involved in the innate immune system.

**Table 2:** List for of proteins classified into IA-2.

Protein name	Gene	Biological function*	Expected location*	Signal peptide*	reference
***IA-2***					
Acyl-CoA-binding protein	DBI	acyl-CoA metabolic process	Extracellular	−	[33]
ADP-ribosylation factor 3	ARF3	ER-Golgi transport	Intracellular	−	[29]
Alpha-(1,3)-fucosyltransferase 6	FUT6	fucosyltransferase activity	Intracellular	−	[30]
Alpha-enolase	ENO1	Glycolytic process	Cytosol	−	[33]
Annexin A1	ANXA1	actin cytoskeleton reorganization	Intracellular, plasma membrane, Extracellular	−	[35]
Annexin A11	ANXA11	actin cytoskeleton reorganization	Intracellular, plasma membrane, Extracellular	−	[33]
Annexin A2	ANXA2	angiogenesis	Intracellular, plasma membrane, Extracellular	−	[33]
Annexin A3	ANXA3	actin cytoskeleton reorganization	Intracellular, plasma membrane, Extracellular	−	[33]
Annexin A5	ANXA5	actin cytoskeleton reorganization	Intracellular, plasma membrane, Extracellular	−	[36]
Apolipoprotein A-I	APOA1	Lipid binding	Secreted, Intracellular	+	[37]
BPI fold-containing family A member 1	BPIFA1	antibacterial humoral response	Secreted, Extracellular	+	[38]
BPI fold-containing family A member 2	BPIFA2	antibacterial humoral response	Secreted, Extracellular	+	[30]
BPI fold-containing family B member 2	BPIFB2	antibacterial humoral response	Secreted, Extracellular	+	[31]
Calmodulin	CALM1	calcium ion binding	Intracellular	−	[33]
Carbonic anhydrase 2	CA2	carbonate dehydratase activity	Cytosol, plasma membrane	−	[29]
Carcinoembryonic antigen-related cell adhesion molecule 8	CD66b	immune response	Integral to plasma membrane	+	[39]
Cathelicidin antimicrobial peptide	CAMP	antibacterial humoral response	Secreted	+	[40]
Chloride intracellular channel protein 1	CLIC1	cytoskeleton organization	Extracellular, plasma membrane	−	[29]
Chloride intracellular channel protein 4	CLIC4	cytoskeleton organization	Extracellular, plasma membrane	−	[29]
Cofilin-1	CFL1	actin cytoskeleton organization	Cytosol, plasma membrane	−	[33]
Cystatin-S	CST4	cysteine-type endopeptidase inhibitor activity	Secreted	+	[30]
Cystatin-SA	CST2	cysteine-type endopeptidase inhibitor activity	Secreted	+	[41]
Cystatin-SN	CST1	cysteine-type endopeptidase inhibitor activity	Secreted	+	[42]
Desmoglein-3	DSG3	Cell adhesion	Integral to plasma membrane	+	[43]
Erythrocyte band 7 integral membrane protein	STOM	protein homooligomerization	Cytosol	−	[29]
Ezrin	EZR	actin cytoskeleton reorganization	Cytoskeleton	−	[33]
Gelsolin	GSN	actin filament reorganization	Intracellular	+	[44]
Glutathione S-transferase P	GSTP1	glutathione transferase activity	Cytosol	−	[33]
Glyceraldehyde-3-phosphate dehydrogenase	GAPDH	Glycolytic process	Cytosol	−	[45]
Guanine nucleotide-binding protein G(I)/G(S)/G(O) subunit gamma-7	GNG7	G-protein coupled receptor signaling pathway	plasma membrane	−	[29]
Heat shock cognate 71 kDa protein	HSPA8	Stress response	Cytosol, Plasma membrane	−	[46]
Kunitz-type protease inhibitor 2	SPINT2	serine-type endopeptidase inhibitor activity	Integral to plasma membrane	+	[31]
Lactoperoxidase	LPO	antibacterial response	Secreted	+	[30]
Lysosome-associated membrane glycoprotein 2	LAMP2	chaperone-mediated autophagy	Integral to Lysosome membrane	+	[28]
Lysozyme C	LYZ	antimicrobial humoral response	Secreted	+	[37]
Moesin	MSN	actin cytoskeleton reorganization	Cytoskeleton	−	[47]
Nucleobindin-2	NUCB2	calcium ion binding	Cytosol, Extracellular	+	[48]
Proactivator polypeptide	PSAP	lipid transport	Secreted	+	[29]
Programmed cell death protein 10	PDCD10	apoptotic process	Cytosol, Plasma membrane	−	[44]
Protein S100-A1	S100A1	calcium ion binding	Cytosol	−	[49]
Protein S100-A11	S100A11	calcium ion binding	Cytosol	−	[29]
Pyruvate kinase isozymes M1/M2	PKM2	Glycolytic process	Cytosol	−	[33]
Rab GDP dissociation inhibitor beta	GDI2	GTPase activation	Cytosol	−	[50]
Radixin	RDX	actin cytoskeleton reorganization	Cytoskeleton	−	[50]
Ras-related C3 botulinum toxin substrate 2	RAC2	actin filament organization	Cytosol	−	[44]
Ras-related protein Rab-3D	RAB3D	Exocytosis pathway	Cytoskeleton, Plasma membrane	−	[33]
Ras-related protein Rab-7a	RAB7A	late endosome transport	Endosome, Lysosome	−	[29]
Ras-related protein Ral-B	RALB	cell migration, cell proliferation	Plasma membrane	−	[33]
Rho-related GTP-binding protein RhoG	RHOG	actin cytoskeleton organization	Plasma membrane	−	[27]
Secreted frizzled-related protein 1	SFRP1	Wnt signaling pathway	Secreted	+	[30]
Serum amyloid A-1 protein	SAA1	acute-phase response	Cytoskeleton, Secreted	+	[33]
Syntaxin-3	STX3	vesicle fusion	Plasma membrane	−	[45]
Thrombospondin-1	THBS1	blood coagulation	Extracellular, Endoplasmic reticulum	+	[29]
Toll-interacting protein	TOLLIP	inflammatory response	Cytosol	−	[33]
Triosephosphate isomerase	TPI1	Glycolytic process	Cytosol	−	[33]
Tumor-associated calcium signal transducer 2	TACSTD2	cell proliferation	Integral to plasma membrane	+	[51]
V-type proton ATPase subunit E 1	ATP6V1E1	proton transport	Extracellular, Cytosol, Plasma membrane	−	[48]
Vesicle-associated membrane protein 8	VAMP8	vesicle fusion	Plasma membrane	−	[37]
Zymogen granule protein 16 homolog B	ZG16B	unknown	Secreted	+	[40]

* Based on Uniprot (https://www.uniprot.org/).

## Discussion

In this study, soluble fractions of human whole saliva after 2,600 g centrifugation were spun at 160,000 g to obtain sediment of particles. Then, these particles were fractionated using density gradient centrifugation, which has often been used to separate vesicular particles (which have low density) from nonvesicular entities, such as supramolecular complex that display high density [4]. The methodology has also been used to subtype EVs based on their slight differences in density [2, 15, 17, 19, 20]. However, the kinetics of particles in media are influenced by the size, shape, and frictional characteristics of particles [52], and therefore it is very difficult to configure the conditions that allow all particles to achieve equilibrium, especially for EV populations that display a wide range of diversity. Standard conditions for density gradient centrifugation (e.g., 160,000 g for 17 h) have been shown to be insufficient for achieving equilibrium [2, 20–22]. In order to determine the innate density of subclasses of EV, a prolonged time (96 h) for density gradient centrifugation was employed in this study. In addition, particles were centrifuged for fractionation both in downward and upward directions, and if a given cargo protein was recovered from the same density fraction irrespective of movement directions, the particle carrying the protein was regarded as being in equilibrium. We triplicated these analyses using whole saliva from three healthy volunteers, and we discovered that 111 proteins (Group I) achieved equilibrium in all three specimens (Fig 3A). By using the total area obtained by MS as the index of quantitative evaluation, the fraction that contained the largest quantity of a given protein was defined as "peak fraction density (PFD)" and was mapped for 333 (111 x 3) proteins (S4 Fig). Patterns of PFD were primarily classified into two types, Group IA and Group IB. Group IB contained immune-globin, HSPB1, S100A14, and other similar proteins (S1 Dataset), and displayed identical density among the three specimens, which was relatively high (1.16 g/ml) compared to the reported density of EVs. With the current available data, we do not know whether the proteins in Group IB are associated with EVs or not. Atomic force microscopy (AFM) observation detected numerous tiny particles in the denser fractions of EVs, which are not protected from protease treatment as in the case of EV-included proteins, suggesting many proteins in the high-density fraction are not included in the cargo of EVs, but are instead the extraneous entities of vesicles [15]. Recent reports by other groups have also concluded that most molecules recovered from high density fractions in density gradient separation were nonvesicular components [4, 18].

Whereas Group IA proteins had identical PFD values, the ones from Group IA exhibited specimen-dependent drifts; that is, each specimen had idiosyncratic PFD values, the patterns of which were classified mainly into two types. One type (Group IA-1) exhibited the pattern of 1.12 g/ml, 1.10 g/ml and 1.09 g/ml, and was composed of 16 proteins, which included classical exosome markers, CD63, CD9 and CD81. This group also contained proteins related to vesicle formation as well as components of the immune system (Table 1). Group IA-2, whose PFD values were 1.12 g/ml, 1.13 g/ml and 1.09 g/ml for Specimens 1, 2 and 3, respectively, was composed of 59 proteins, and many of them are involved in membrane dynamics and the innate immune system (Table 2). IA-2 also contained many proteins responsible for this innate pathogen protecting system, including lysozyme C [53], lactoperoxidase [54], cystatin S, cystatin SA, and cystatin SN [55], BPI fold-containing families (BPIFA1, BPIFA2, BPIFB2) [56], cathelicidin antimicrobial peptide (CAMP) [57], and toll-interacting protein (TOLLIP) [58] (Table 2). Interestingly, it has been suggested that lysozyme, peroxidase, and lactoferrin are secreted from the acinic cells of the salivary gland via exocytosis transport as free proteins [59]. Our observation suggested the following possibilities: 1) secretory vesicles that contain these proteins are released from acinic cells; or 2) these proteins attached themselves to the exterior side of EVs in saliva after they are released as free form proteins. It is also noteworthy that Group IA-2 contained some of glycolytic enzymes; that is, GAPDH, TPI1, ENO1, PKM2, PGAM1, PGK1, and ALDOA (Table 2). Further studies including morphological observation are required to determine the role of these glycolytic enzymes in EVs; however, some of these enzymes have been shown to regulate membrane dynamics [60, 61] and some of them have been reported to be recovered from extracellular space [4].

Our study also identified 21 proteins that did not attain a state of equilibrium in any of the three specimens under the conditions used (Group II). They included a -amylase, apolipoprotein (a) and others, which may have particular forms that retard their movement in media. The results emphasize the importance of two direction (upward and downward) analyses in density-gradient centrifugation. The list of proteins in Group II is found in S1 Dataset.

To elucidate the molecular mechanism that causes specimen-specific density drift, further studies are required; however, it is possible that environments of oral cavity (such as pH or osmotic pressure) could alter the physiochemical properties of particular subclasses of EV that have certain channel proteins. In this regard, it is noteworthy that the osmotic pressure of saliva is correlated with the rate of saliva secretion (or the amount of secretion) [62]. It should be interesting to investigate the effects of forced environmental disturbances on the properties of subsets of EVs *in vitro*.

The observations obtained here would offer new insight into EV-biology and contribute to the development of salivary EV diagnostic systems. The 111 proteins contained diagnostic markers, such as SLC44A4 [63], PMSA [64], CEACAM8/CD66b [65], and Serum amyloid A-1 protein (SAA1) [66]. Because these diagnostic markers have a probability of coboarding with the other proteins within the same subgroup, the information obtained in this study would help to select coboarded molecules that are used in sandwich ELISA [67] or affinity-based preconcentration [10–12].

## Supporting information

S1 Fig The effects of a sonicating pretreatment of whole saliva on the properties of crude EV fraction. (A) Thirty mL of whole saliva from a healthy volunteer was divided into two portions, one of which was sonicated as shown in the Material and methods section. The larger materials were removed by centrifuging at 2,600 g for 30 min at 4°C, and the resultant supernatants were centrifuged at 100,000 g for 110 min at 4°C (Avanti JXN-30 with JS-24.38 rotor, Beckman Coulter (equivalent to 160,000 g for 70 min)) to obtain the crude 500 μL of crude EV fraction. (B) Ten μL of the crude EV fraction were placed on formvar-coated (Nissin EM, Tokyo, Japan) nickel grids (S-300 square mash, Gilder, Grantham, UK). After staining with 2% uranyl acetate (Wako, Tokyo, Japan) for 1 min, transmission electron microscopy (TEM) images were obtained with a H-7650 instrument (Hitachi, Co., Tokyo, Japan). (C) Aliquots of the samples were smeared on glass slides (Superfrost, Matsunami Glass Inc., Osaka, Japan) and stained with Papanicolaou’s solution. Optical microscopic images were taken using a 100X object lens (Plan Apochromat, 1.45 x 0.13 mm, Keyence, Osaka, Japan) equipped on a BZ-X800 (Keyence), and the images were processed in Z-stack mode by using a BZ-800 Analyzer ver.1.1.2; the bars represent 100 μm. (D) The western blot experiment was performed as described in the Material and methods section. The antibody used was mouse anti-CD81 (SHI-EXO-M03, Cosmobio, Tokyo, Japan; 1:1,000 dilution). Both sonicated and non-sonicated (sonic, + and–in the figure) samples were run in the same gel and blotted on the same membrane, as shown below. (E) The number and size distributions of the particles in the crude EV fractions were evaluated by the nanoparticle tracking analysis (NTA) method using the NanoSight LM10 system and NTA software version 2.3 (Malvern Instruments Ltd., Worcestershire, UK). Silica beads (diameter:100 nm) were used in calibration (24041, Polysciences, PA, USA), and the camera level (CL) and detection threshold (DT) were set at values of CL 14 and DT 4. For each sample, measurements (30 s) were independently performed five times.
(PDF)

S2 Fig Silver staining analyses of each fraction. Ten fractions (F1 to F10) obtained in downward (D) and upward (U) fractionation and for 17 h and 96 h of centrifugation were run through SDS-PAGE, and their contents were visualized using silver staining. For the 96 h centrifugation, 10% gel was used, and for the 17-h centrifugation, 15% gel was used. The sample for 96 h was identical to Specimen 1, and the one for 17 h was independently prepared from the identical individual but on a different day. Molecular weights of markers are indicated on both sides and the measured densities are indicated. Green broken lines highlight non-equilibrium state of molecules in 17 h centrifugation.
(PDF)

S3 Fig WB analyses of fractionated human saliva. The WB results for (in descending order): CD63, CD81, CD9, α-amylase, HSP70, AQP5 and TSG101 are shown. For each protein, the top row is for the downward separation, and the bottom is for the upward separation. The three columns on the left side of the dotted line are for the 96-h centrifugation for (L–R) Specimen 1, Specimen 2, and Specimen 3. Molecular weight markers are indicated on the left of each panel, and the fraction numbers and their measured densities (g/ml) are indicated at the top of each panel. The Antibodies used and their dilution rates in WB were the following; mouse anti-CD63 (ab8219, Abcam, Cambridge, UK; 1:1,000); mouse anti-CD81 (11-558-C100, EXBIO, Praha, a.s., Vestec, Czech Republic; 1:1,000); mouse anti-CD9 (ab124476, Abcam; 1:500, for specimen 1); anti-CD9 (SHI-EXO-M01, Cosmobio, Tokyo, Japan; 1:1,000, for Specimen 2 and Specimen 3); mouse anti-alpha-amylase (ab54765, Abcam; 1:400), rabbit anti-HSP70 (EXOAB-Hsp70A-1, System Biosciences LLC, Palo Alto, CA, USA; 1:1,000); rabbit anti-aquaporin 5 (AQP5) (ab92320, Abcam; 1:500), and mouse anti-TSG101 (612969, BD Transduction Laboratories, Franklin Lakes, NJ, USA; 1:500), Secondary antibodies coupled to horseradish peroxidase included the following: goat anti-mouse IgG (H + L)-HRP conjugate (170–6516, Bio-Rad, Hercules, CA, USA; 1:2,000) and goat anti-rabbit IgG (H + L)-HRP conjugate (170–6515, Bio-Rad; 1:2,000). For reference, the results from the fractions of the 17 h centrifugation, which were independently prepared from the identical individual for Specimen 1 but on a different day, are shown on the right side. For the 17 h samples, only CD63, CD81, α-amylase, HSP70 and AQP5 were tested.
(PDF)

S4 Fig Heat map representations of protein quantities in ten fractions of proteins belonging to the IA-1 and IA-2 subgroups. Relative amounts of the protein found in each fraction were estimated from the total area obtained by MS and are plotted against densities in the heat map for Specimen 1 (S.1), Specimen 2 (S.2), and Specimen 3 (S.3).; the logarithm values in a unit were normalized by the maximum. The fraction with the maximum was colored with red. According to the amount of each protein detected in every unit, heat maps were drawn from red to green using 256 steps in SVG format. If the protein was not detected, the corresponding fraction was colored with gray. Whereas experimentally 10 fractions were available, their densities varied depending on the units of samples. As illustrated in Fig 2, F1 to F10 were spread out actual density fractions, which resulted in many gray lanes appearing in the heat maps. The Perl script used in these analyses is available from GitHub repository (https://github.com/yamamoto-tdc/EV-saliva). CD63 is shown in the top of the figure as the representative. D and U denote downward and upward separation, respectively.
(PDF)

S1 Dataset A list of proteins found from human whole saliva in Excel data format. Data 1 column shows specimen numbers in which the protein was identified in no less than one from 20 fractions (10 fractions x two directions). Data 2 column represents the subclasses of the protein (see text). Density values of PFD (see text) for each specimen are shown for three specimens in the right three columns, in which "X" indicates that the protein was not detected from either or both directions, and "N. E." denotes that the protein has not achieved equilibrium in the specimen.
(XLSX)

S2 Dataset These Excel data are raw MS area files about specimen 1, 2 and 3 (each upward and downward). (ZIP)

S1 Video Reduced viscosity of the crude EV fraction by sonication. The pellets of the crude extraction fractions were resuspended in PBS by pipetting. Without the sonication pre-treatment (left), the pellet was very viscous, whereas with sonication (right), it was less viscous.
(MP4)

S1 File (PDF)
