# Supplementary figures and images for: Specimen-specific drift of densities defines distinct subclasses of extracellular vesicles from human whole saliva
Source: PLoS One. 2021 Apr 8;16(4):e0249526. doi: 10.1371/journal.pone.0249526 (PMC8032098; doi:10.1371/journal.pone.0249526)

A

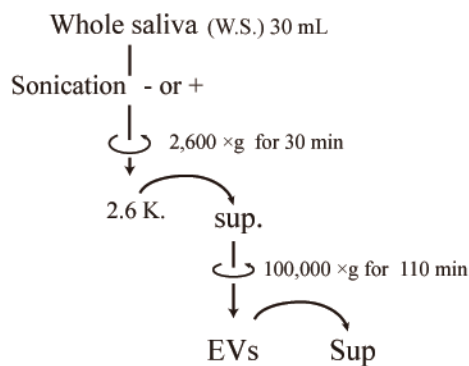

B

Non Sonicated EVs

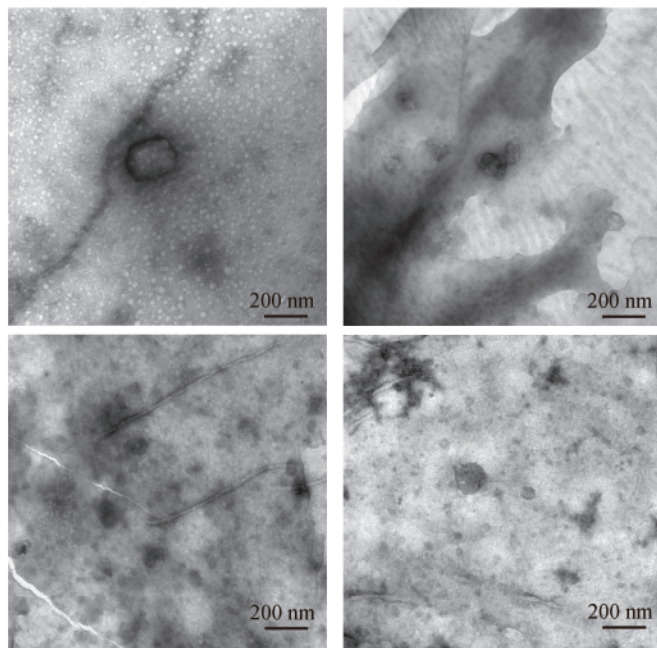

Sonicated EVs

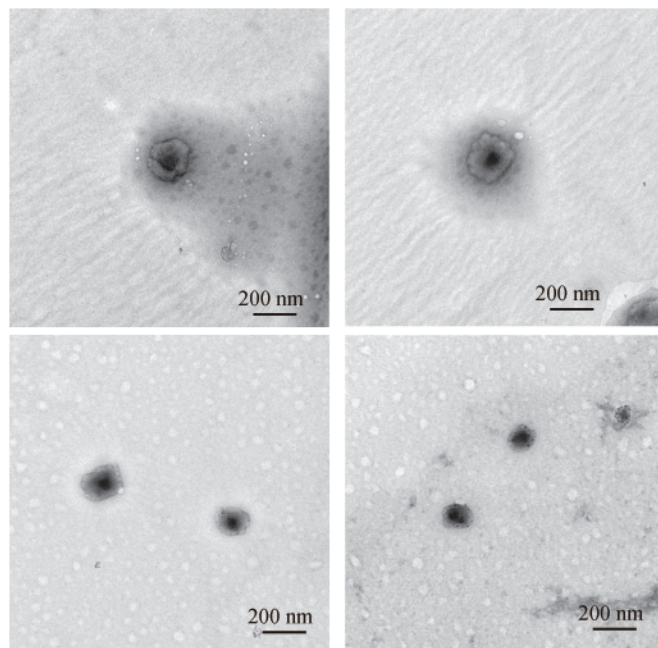

C

Non Sonicated EVs

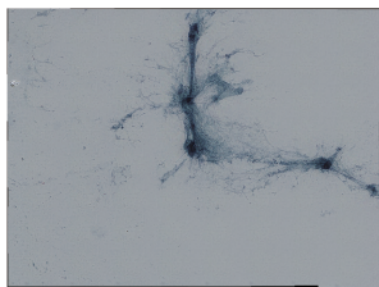

Sonicated EVs

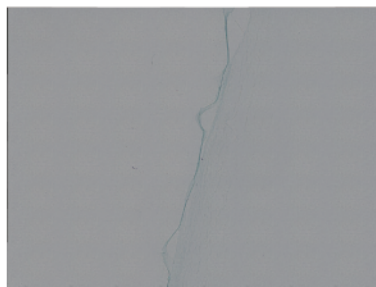

D

CD81

sonication

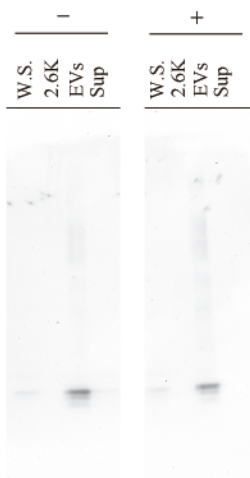

E

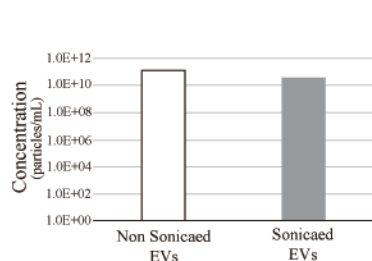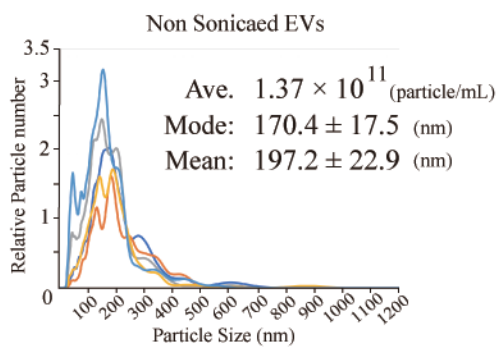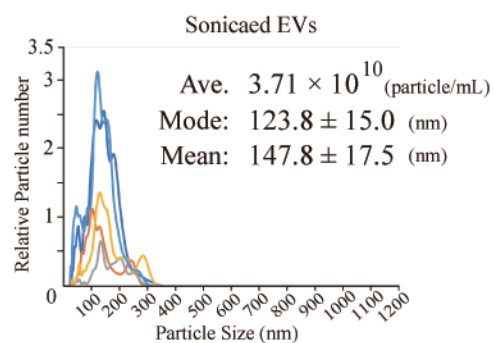

Supplement: S1 Fig — (A) Thirty mL of whole saliva from a healthy volunteer was divided into two portions, one of which was sonicated as shown in the Material and methods section. The larger materials were removed by centrifuging at 2,600 g for 30 min at 4°C, and the resultant supernatants were centrifuged at 100,000 g for 110 min at 4°C (Avanti JXN-30 with JS-24.38 rotor, Beckman Coulter (equivalent to 160,000 g for 70 min)) to obtain the crude 500 μL of crude EV fraction. (B) Ten μL of the crude EV fraction were placed on formvar-coated (Nissin EM, Tokyo, Japan) nickel grids (S-300 square mash, Gilder, Grantham, UK). After staining with 2% uranyl acetate (Wako, Tokyo, Japan) for 1 min, transmission electron microscopy (TEM) images were obtained with a H-7650 instrument (Hitachi, Co., Tokyo, Japan). (C) Aliquots of the samples were smeared on glass slides (Superfrost, Matsunami Glass Inc., Osaka, Japan) and stained with Papanicolaou’s solution. Optical microscopic images were taken using a 100X object lens (Plan Apochromat, 1.45 x 0.13 mm, Keyence, Osaka, Japan) equipped on a BZ-X800 (Keyence), and the images were processed in Z-stack mode by using a BZ-800 Analyzer ver.1.1.2; the bars represent 100 μm. (D) The western blot experiment was performed as described in the Material and methods section. The antibody used was mouse anti-CD81 (SHI-EXO-M03, Cosmobio, Tokyo, Japan; 1:1,000 dilution). Both sonicated and non-sonicated (sonic, + and–in the figure) samples were run in the same gel and blotted on the same membrane, as shown below. (E) The number and size distributions of the particles in the crude EV fractions were evaluated by the nanoparticle tracking analysis (NTA) method using the NanoSight LM10 system and NTA software version 2.3 (Malvern Instruments Ltd., Worcestershire, UK). Silica beads (diameter:100 nm) were used in calibration (24041, Polysciences, PA, USA), and the camera level (CL) and detection threshold (DT) were set at values of CL 14 and DT 4. For each sample, measure [file pone.0249526.s001.pdf]

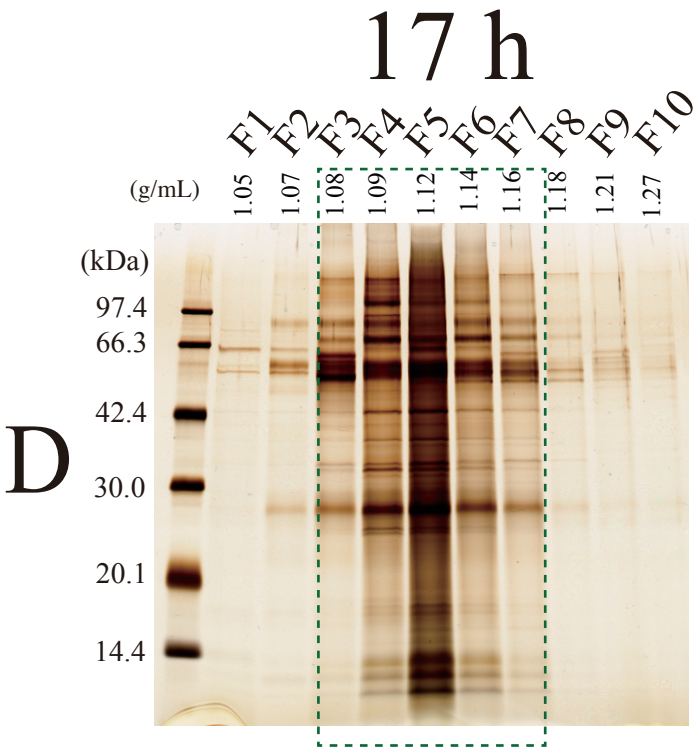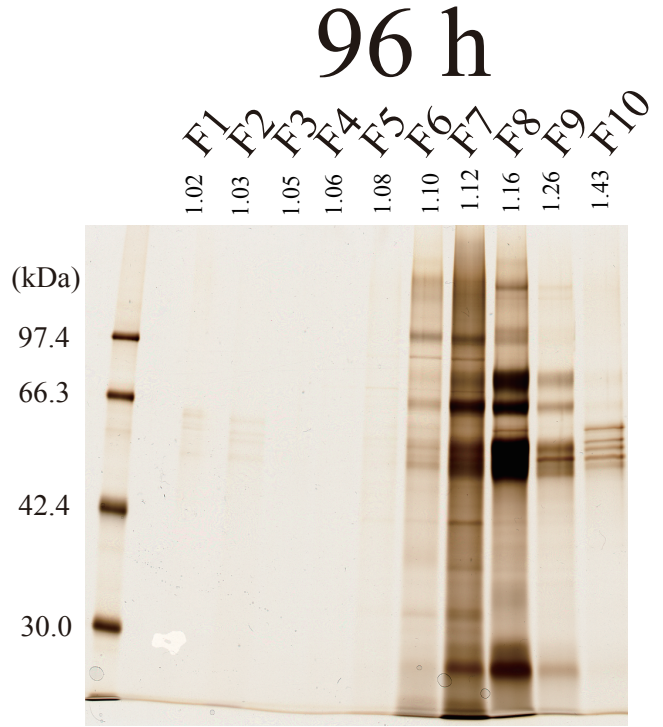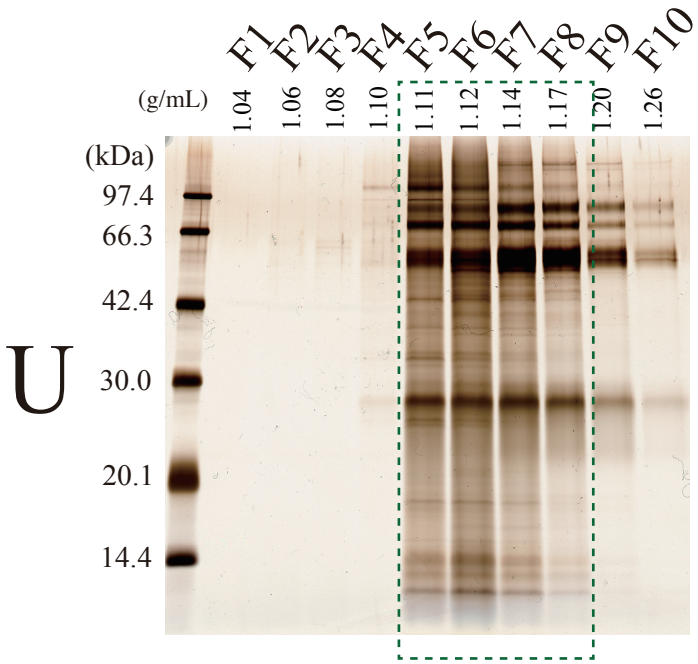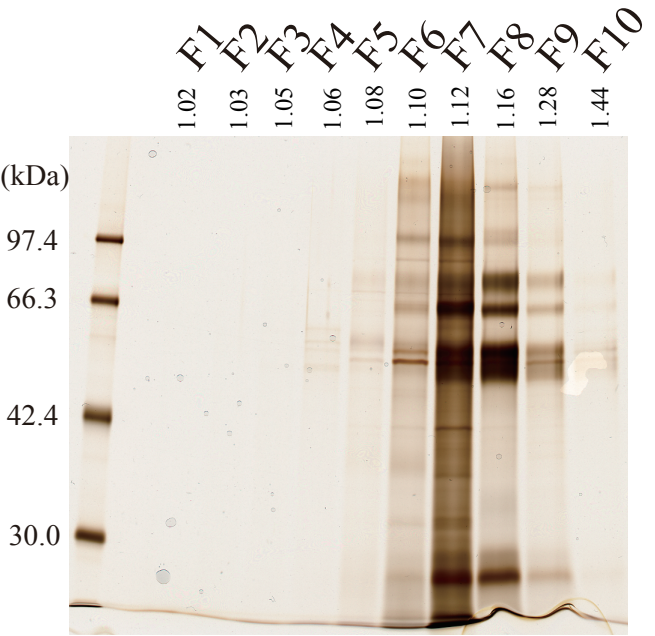

Supplement: S2 Fig — Ten fractions (F1 to F10) obtained in downward (D) and upward (U) fractionation and for 17 h and 96 h of centrifugation were run through SDS-PAGE, and their contents were visualized using silver staining. For the 96 h centrifugation, 10% gel was used, and for the 17-h centrifugation, 15% gel was used. The sample for 96 h was identical to Specimen 1, and the one for 17 h was independently prepared from the identical individual but on a different day. Molecular weights of markers are indicated on both sides and the measured densities are indicated. Green broken lines highlight non-equilibrium state of molecules in 17 h centrifugation. (PDF) [file pone.0249526.s002.pdf]

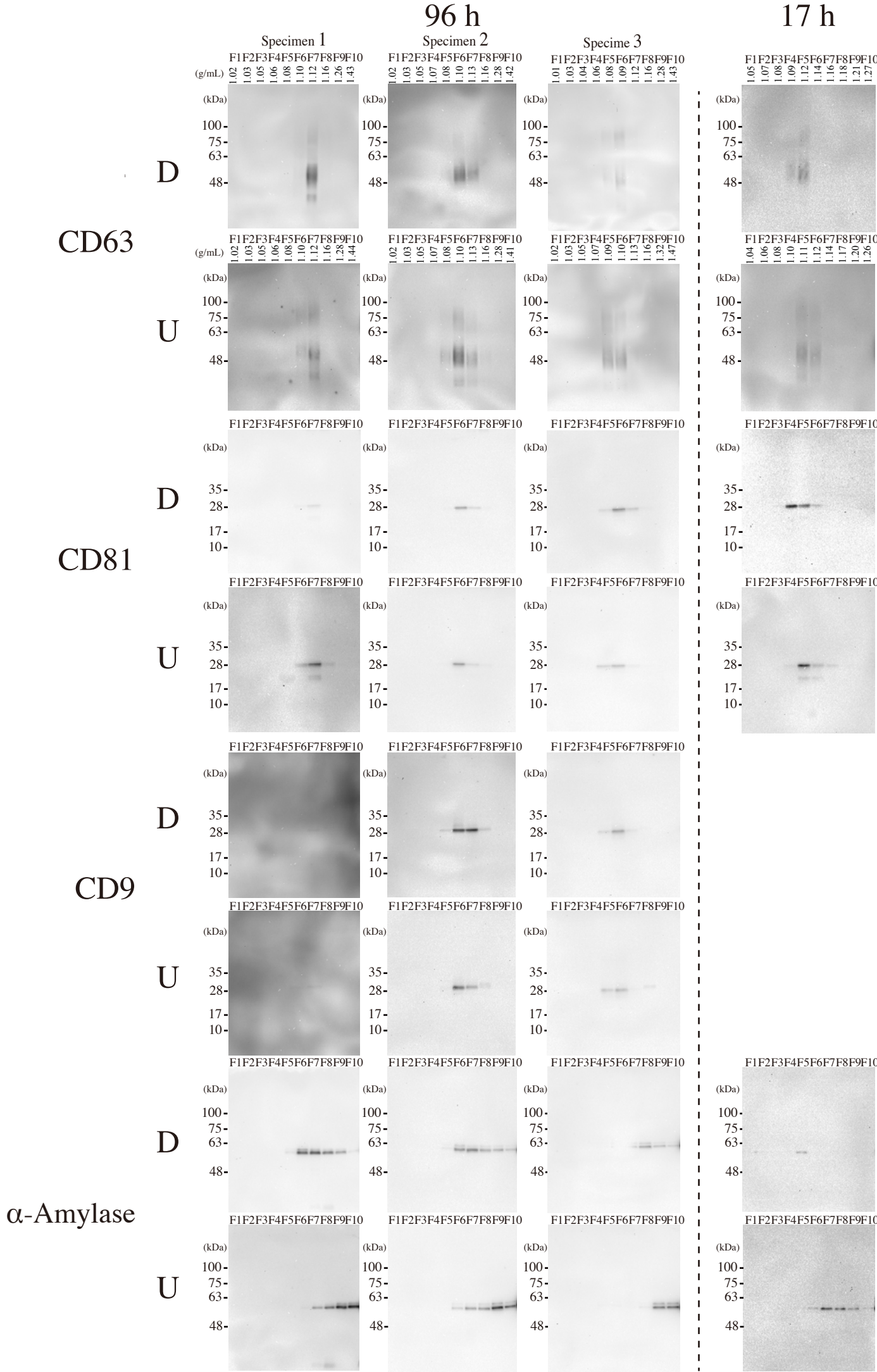

96 h

17 h

Specimen 1

Specimen 2

Specimen 3

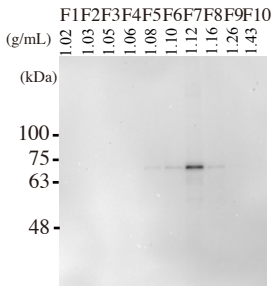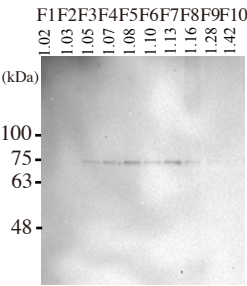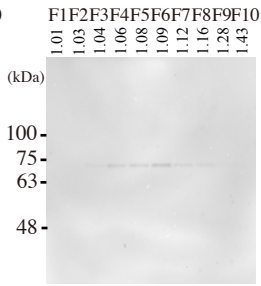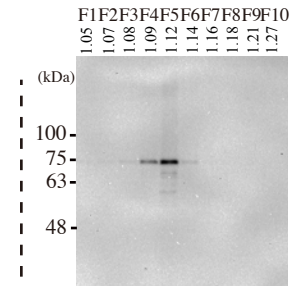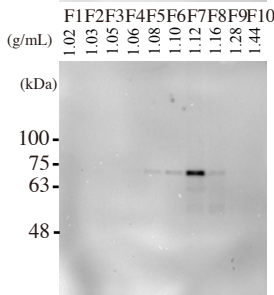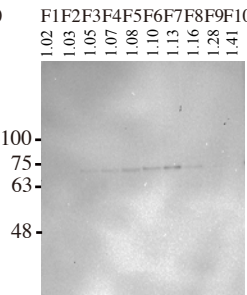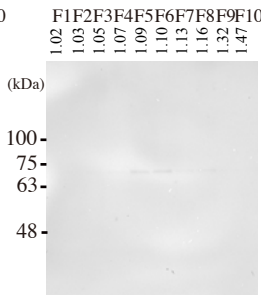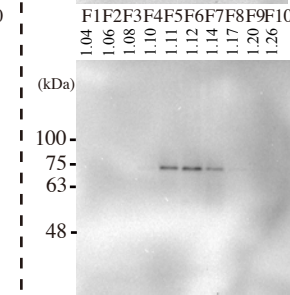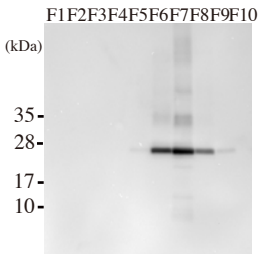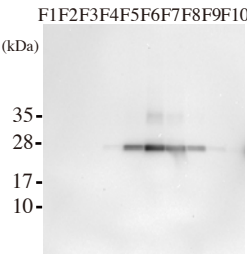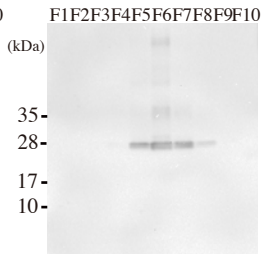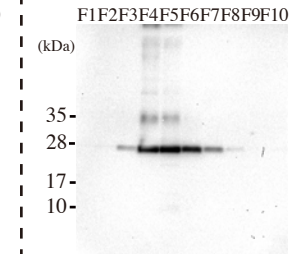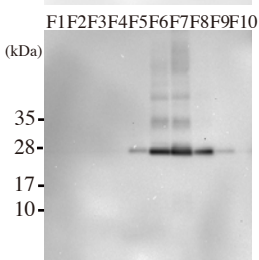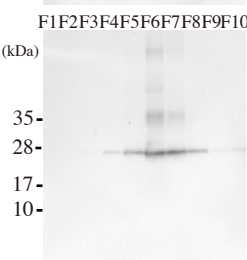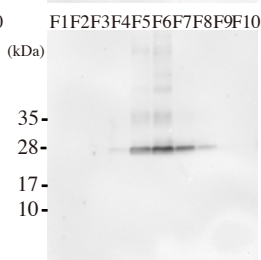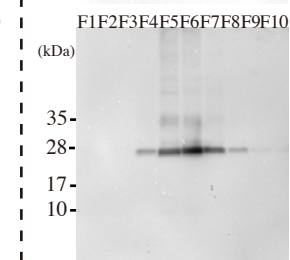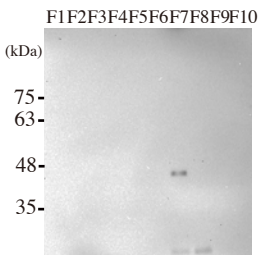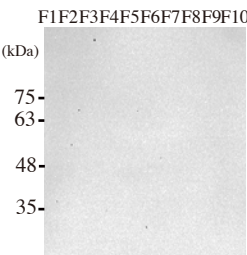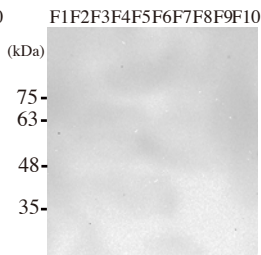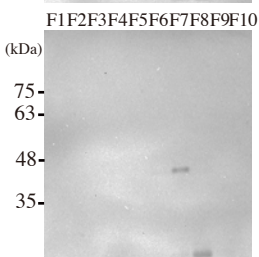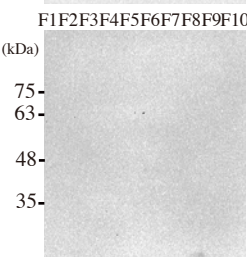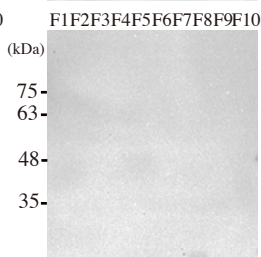

D

U

D

U

D

U

HSP70

AQP5

TSG101

Supplement: S3 Fig — The WB results for (in descending order): CD63, CD81, CD9, α-amylase, HSP70, AQP5 and TSG101 are shown. For each protein, the top row is for the downward separation, and the bottom is for the upward separation. The three columns on the left side of the dotted line are for the 96-h centrifugation for (L–R) Specimen 1, Specimen 2, and Specimen 3. Molecular weight markers are indicated on the left of each panel, and the fraction numbers and their measured densities (g/ml) are indicated at the top of each panel. The Antibodies used and their dilution rates in WB were the following; mouse anti-CD63 (ab8219, Abcam, Cambridge, UK; 1:1,000); mouse anti-CD81 (11-558-C100, EXBIO, Praha, a.s., Vestec, Czech Republic; 1:1,000); mouse anti-CD9 (ab124476, Abcam; 1:500, for specimen 1); anti-CD9 (SHI-EXO-M01, Cosmobio, Tokyo, Japan; 1:1,000, for Specimen 2 and Specimen 3); mouse anti-alpha-amylase (ab54765, Abcam; 1:400), rabbit anti-HSP70 (EXOAB-Hsp70A-1, System Biosciences LLC, Palo Alto, CA, USA; 1:1,000); rabbit anti-aquaporin 5 (AQP5) (ab92320, Abcam; 1:500), and mouse anti-TSG101 (612969, BD Transduction Laboratories, Franklin Lakes, NJ, USA; 1:500), Secondary antibodies coupled to horseradish peroxidase included the following: goat anti-mouse IgG (H + L)-HRP conjugate (170–6516, Bio-Rad, Hercules, CA, USA; 1:2,000) and goat anti-rabbit IgG (H + L)-HRP conjugate (170–6515, Bio-Rad; 1:2,000). For reference, the results from the fractions of the 17 h centrifugation, which were independently prepared from the identical individual for Specimen 1 but on a different day, are shown on the right side. For the 17 h samples, only CD63, CD81, α-amylase, HSP70 and AQP5 were tested. (PDF) [file pone.0249526.s003.pdf]

**For Figure 2 A**

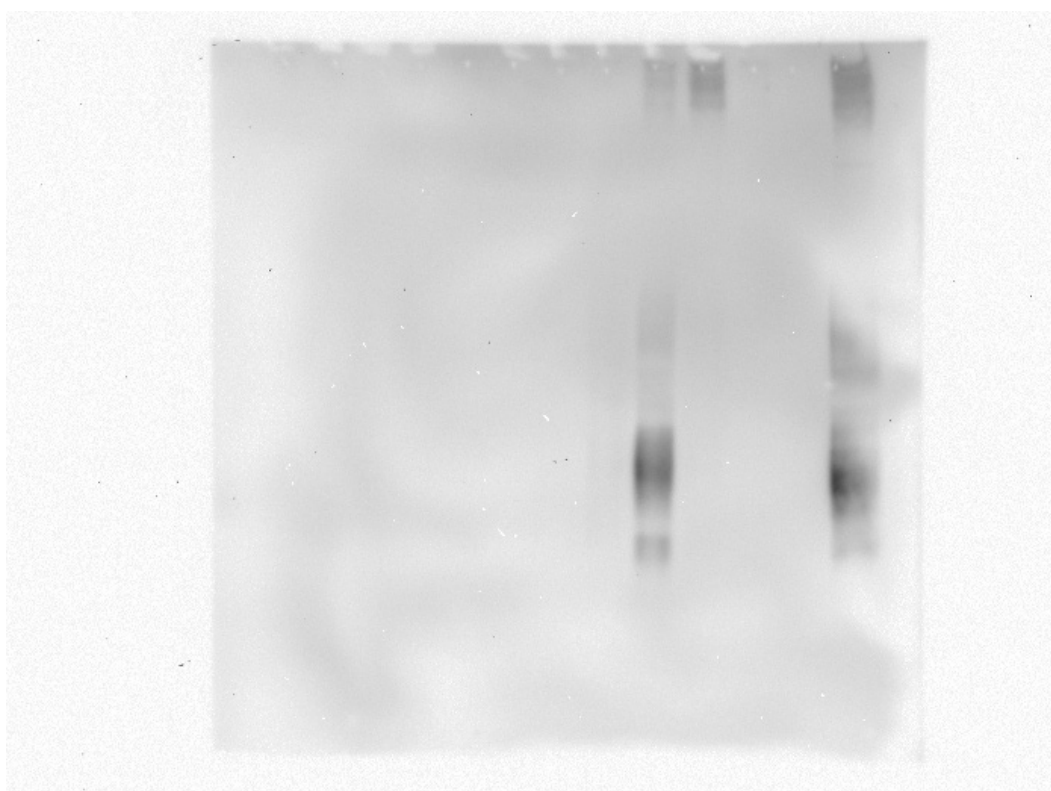

**S.1 D**

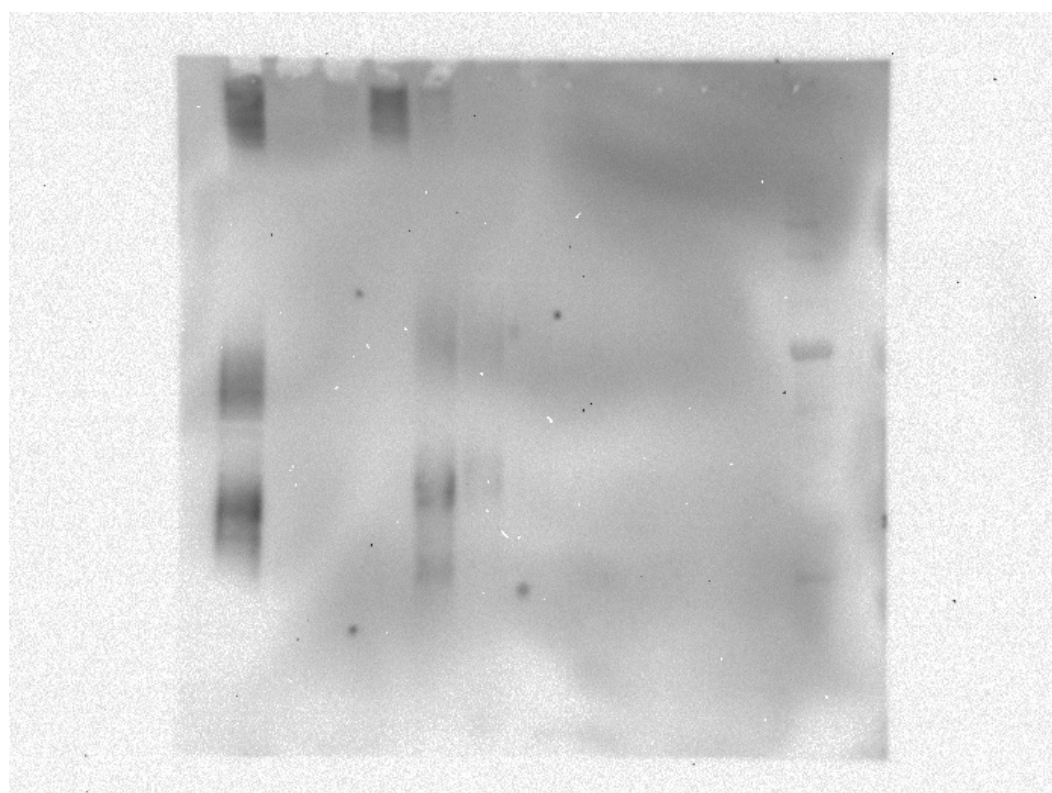

**S.1 U**

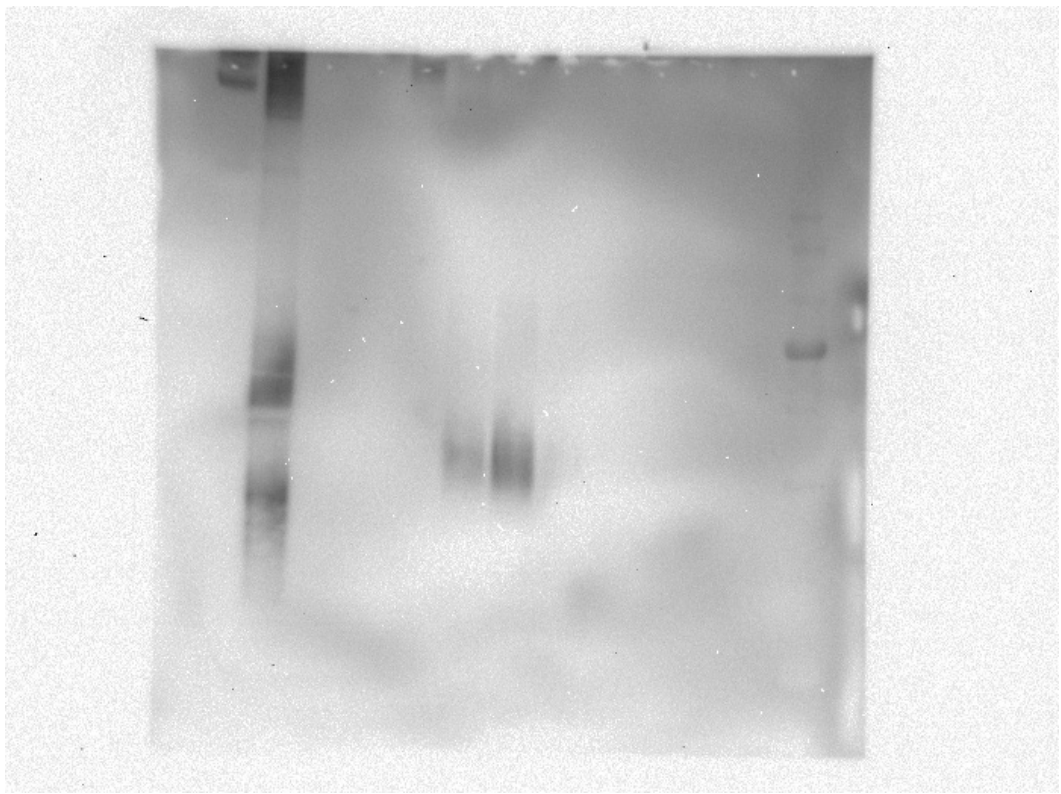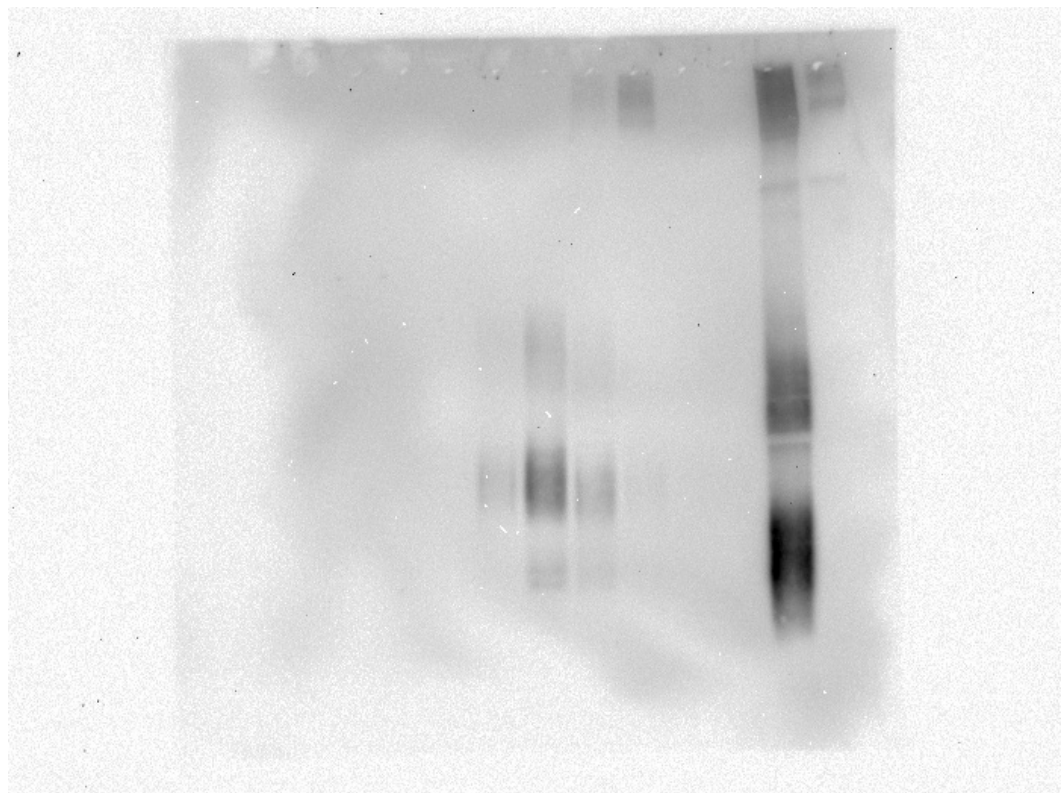

S.2 U

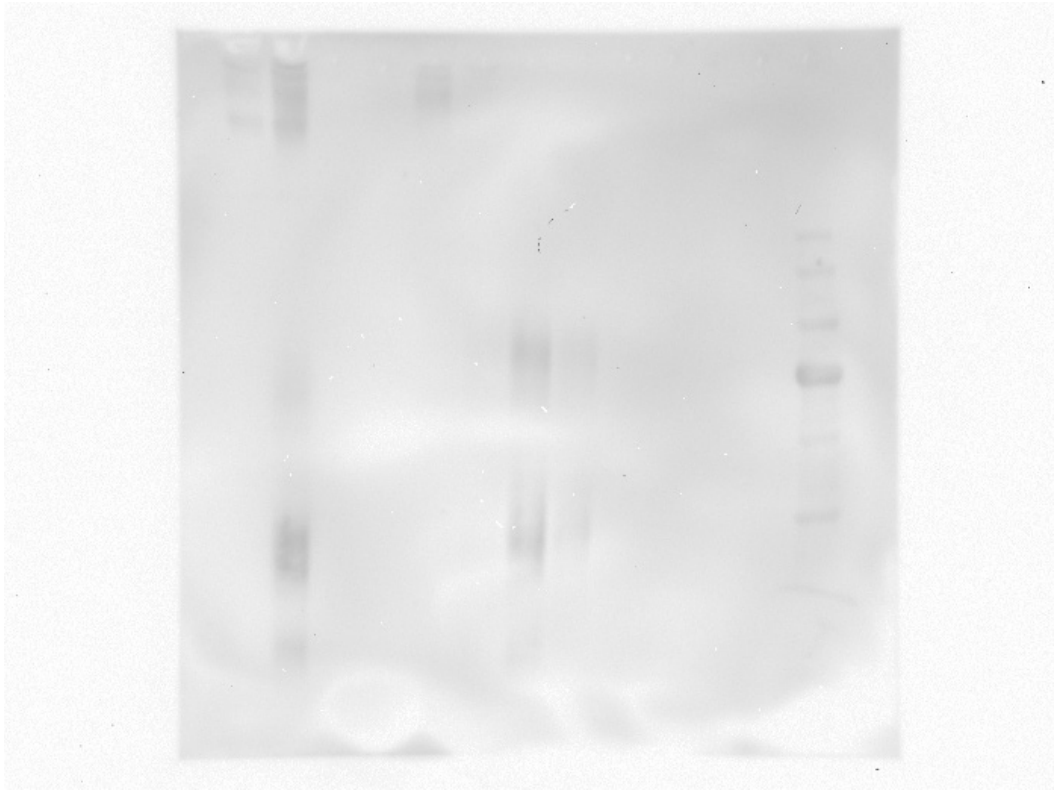

**S.3 D**

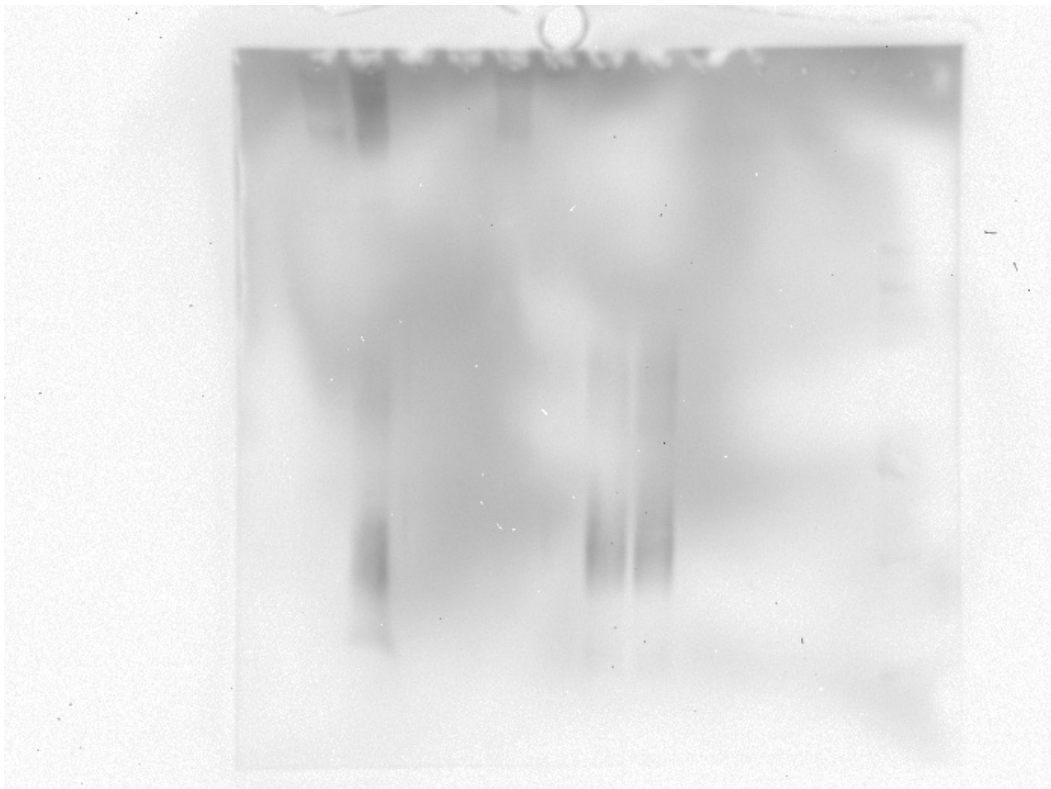

**S.3 U**

**For S1 Fig D**

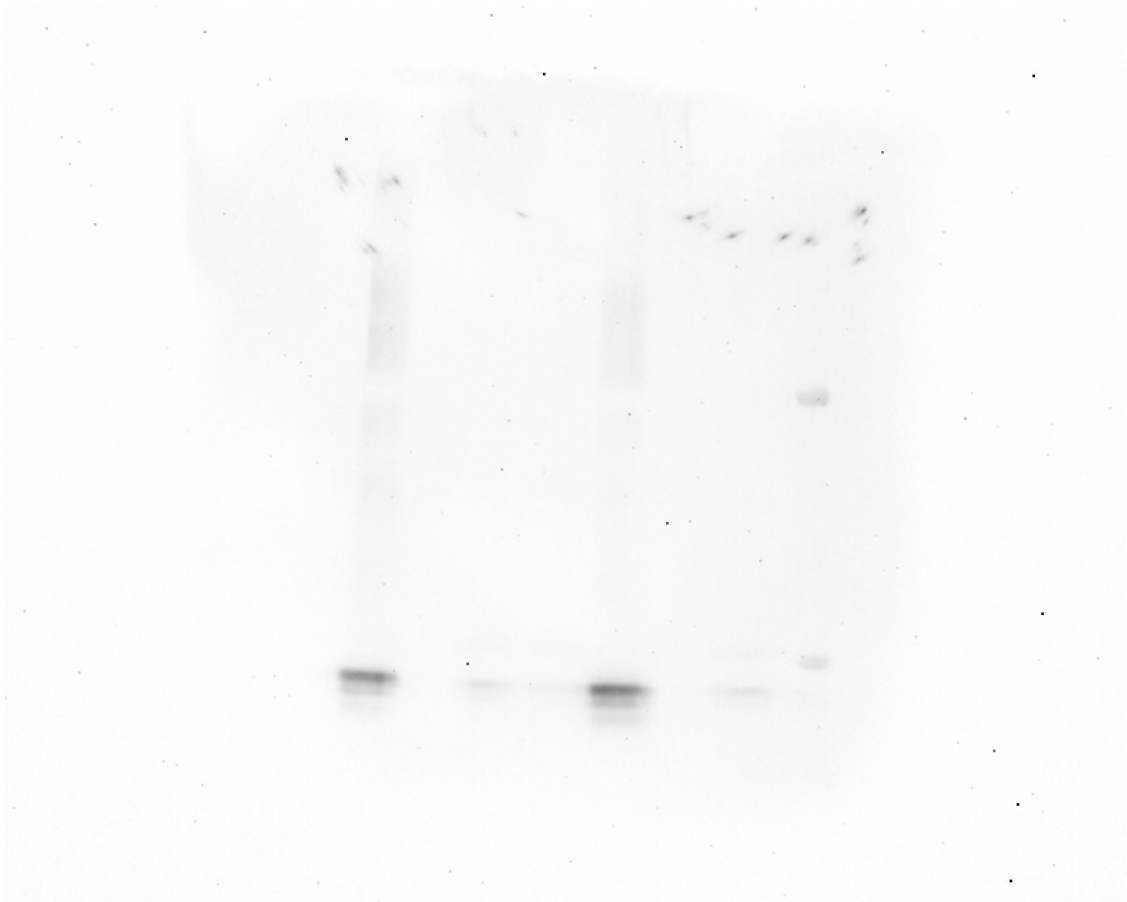

**In S3 Fig, original images are presented.**

Supplement: S1 File — (PDF) [file pone.0249526.s008.pdf]
