# Supplementary material for: Specimen-specific drift of densities defines distinct subclasses of extracellular vesicles from human whole saliva
Source: PLoS One. 2021 Apr 8;16(4):e0249526. doi: 10.1371/journal.pone.0249526 (PMC8032098; doi:10.1371/journal.pone.0249526)
Supplement: S4 Fig — Relative amounts of the protein found in each fraction were estimated from the total area obtained by MS and are plotted against densities in the heat map for Specimen 1 (S.1), Specimen 2 (S.2), and Specimen 3 (S.3).; the logarithm values in a unit were normalized by the maximum. The fraction with the maximum was colored with red. According to the amount of each protein detected in every unit, heat maps were drawn from red to green using 256 steps in SVG format. If the protein was not detected, the corresponding fraction was colored with gray. Whereas experimentally 10 fractions were available, their densities varied depending on the units of samples. As illustrated in Fig 2, F1 to F10 were spread out actual density fractions, which resulted in many gray lanes appearing in the heat maps. The Perl script used in these analyses is available from GitHub repository (https://github.com/yamamoto-tdc/EV-saliva). CD63 is shown in the top of the figure as the representative. D and U denote downward and upward separation, respectively. (PDF) [file pone.0249526.s004.pdf]

IA-1

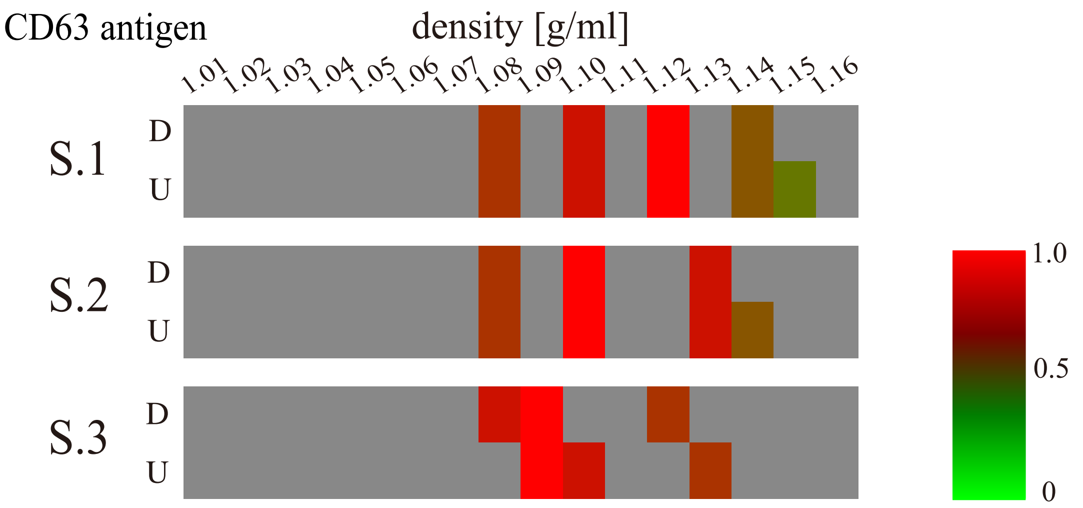
CD63 antigen
density [g/ml]
1.01 1.02 1.03 1.04 1.05 1.06 1.07 1.08 1.09 1.10 1.11 1.12 1.13 1.14 1.15 1.16
S.1
D
U
S.2
D
U
S.3
D
U
1.0
0.5
0

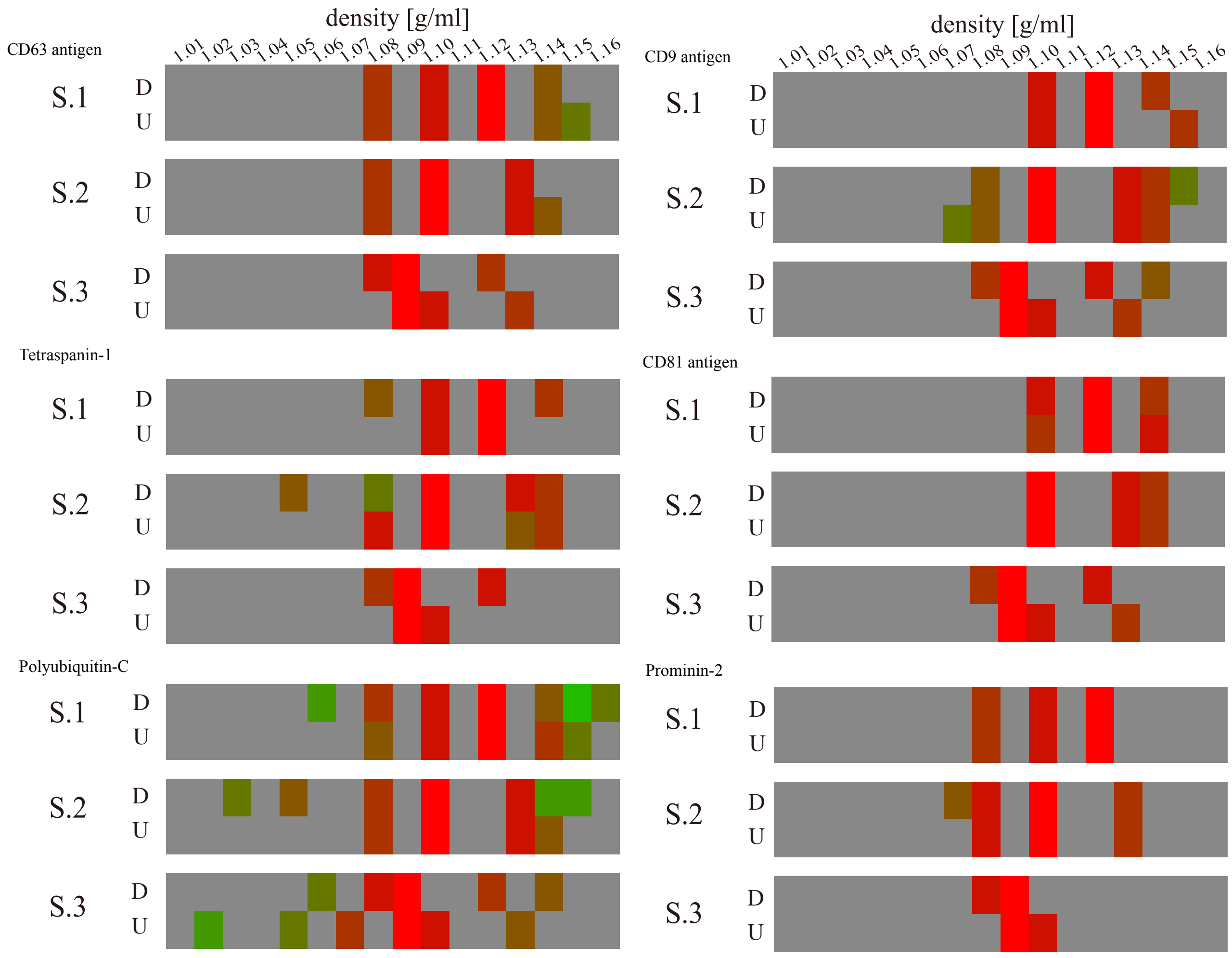
density [g/ml]
CD63 antigen
1.01 1.02 1.03 1.04 1.05 1.06 1.07 1.08 1.09 1.10 1.11 1.12 1.13 1.14 1.15 1.16
S.1
D
U
S.2
D
U
S.3
D
U
Tetraspanin-1
S.1
D
U
S.2
D
U
S.3
D
U
Polyubiquitin-C
S.1
D
U
S.2
D
U
S.3
D
U
density [g/ml]
CD9 antigen
1.01 1.02 1.03 1.04 1.05 1.06 1.07 1.08 1.09 1.10 1.11 1.12 1.13 1.14 1.15 1.16
S.1
D
U
S.2
D
U
S.3
D
U
CD81 antigen
S.1
D
U
S.2
D
U
S.3
D
U
Prominin-2
S.1
D
U
S.2
D
U
S.3
D
U

IA-1

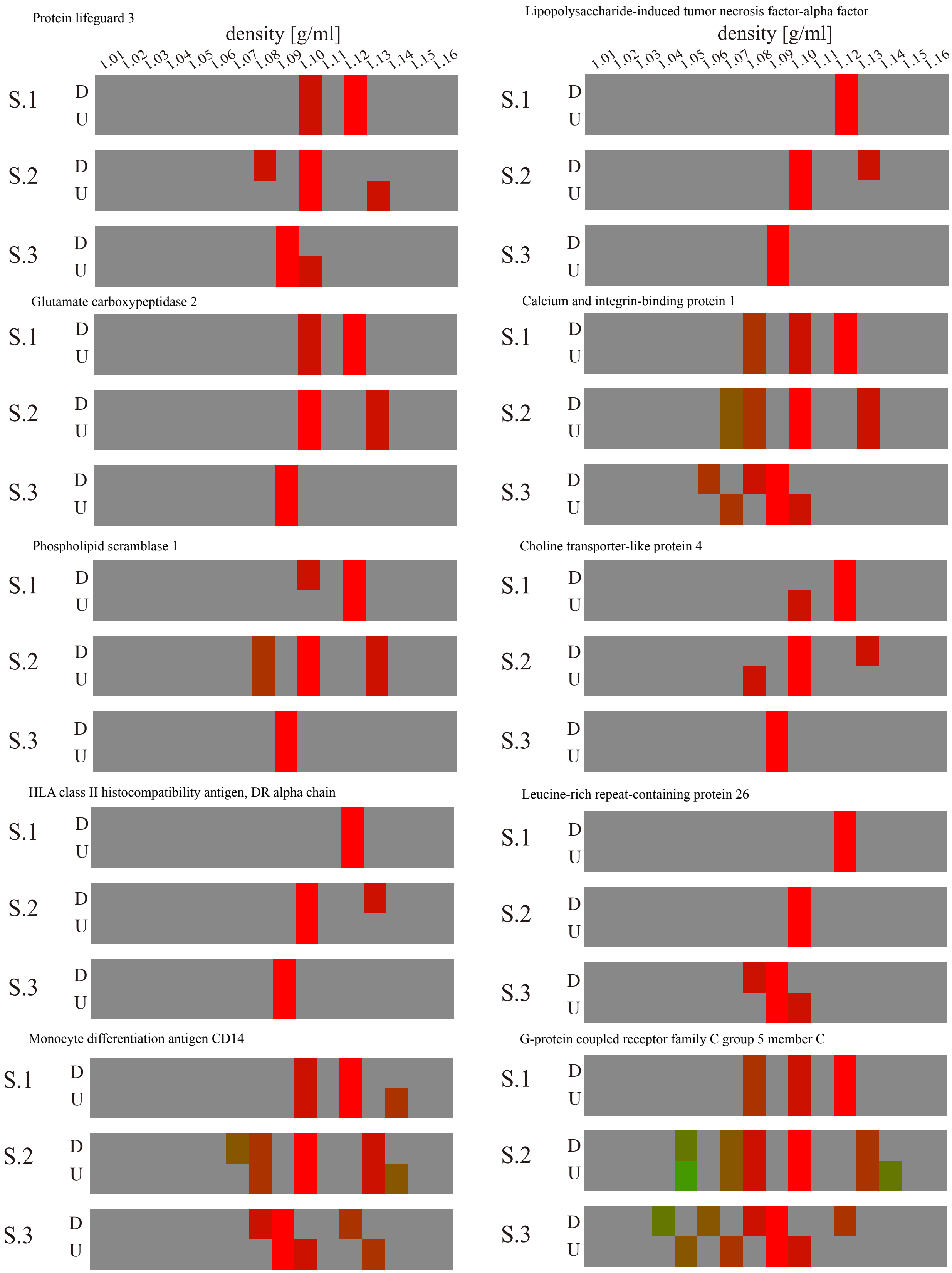

Protein lifeguard 3
Lipopolysaccharide-induced tumor necrosis factor-alpha factor
density [g/ml]
1.01 1.02 1.03 1.04 1.05 1.06 1.07 1.08 1.09 1.10 1.11 1.12 1.13 1.14 1.15 1.16
S.1
S.2
S.3
D
U
Glutamate carboxypeptidase 2
Calcium and integrin-binding protein 1
Phospholipid scramblase 1
Choline transporter-like protein 4
HLA class II histocompatibility antigen, DR alpha chain
Leucine-rich repeat-containing protein 26
Monocyte differentiation antigen CD14
G-protein coupled receptor family C group 5 member C

IA-2

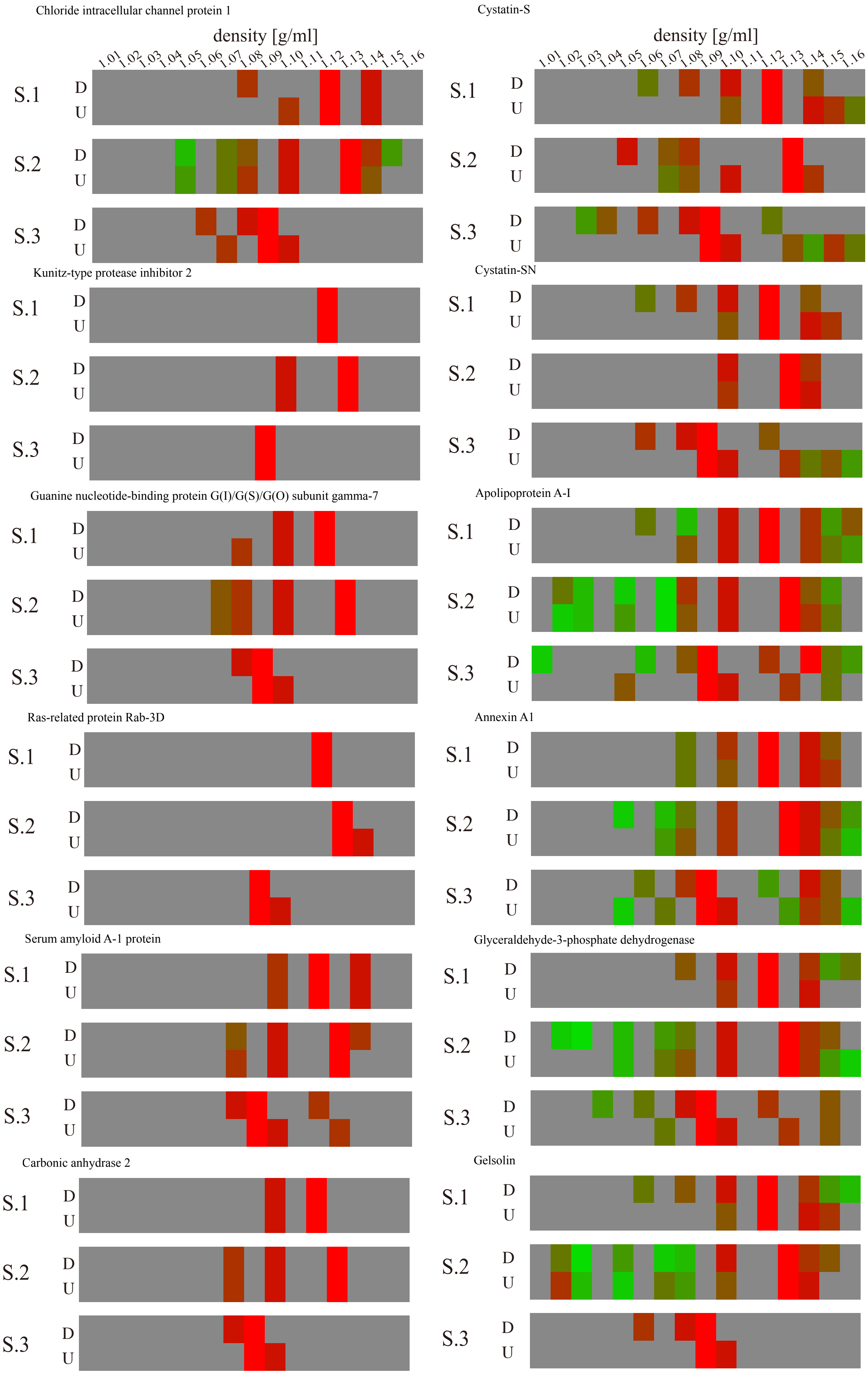
Chloride intracellular channel protein 1
Cystatin-S
density [g/ml]
1.01 1.02 1.03 1.04 1.05 1.06 1.07 1.08 1.09 1.10 1.11 1.12 1.13 1.14 1.15 1.16
S.1
S.2
S.3
D
U
Kunitz-type protease inhibitor 2
Cystatin-SN
Guanine nucleotide-binding protein G(I)/G(S)/G(O) subunit gamma-7
Apolipoprotein A-I
Ras-related protein Rab-3D
Annexin A1
Serum amyloid A-1 protein
Glyceraldehyde-3-phosphate dehydrogenase
Carbonic anhydrase 2
Gelsolin

IA-2

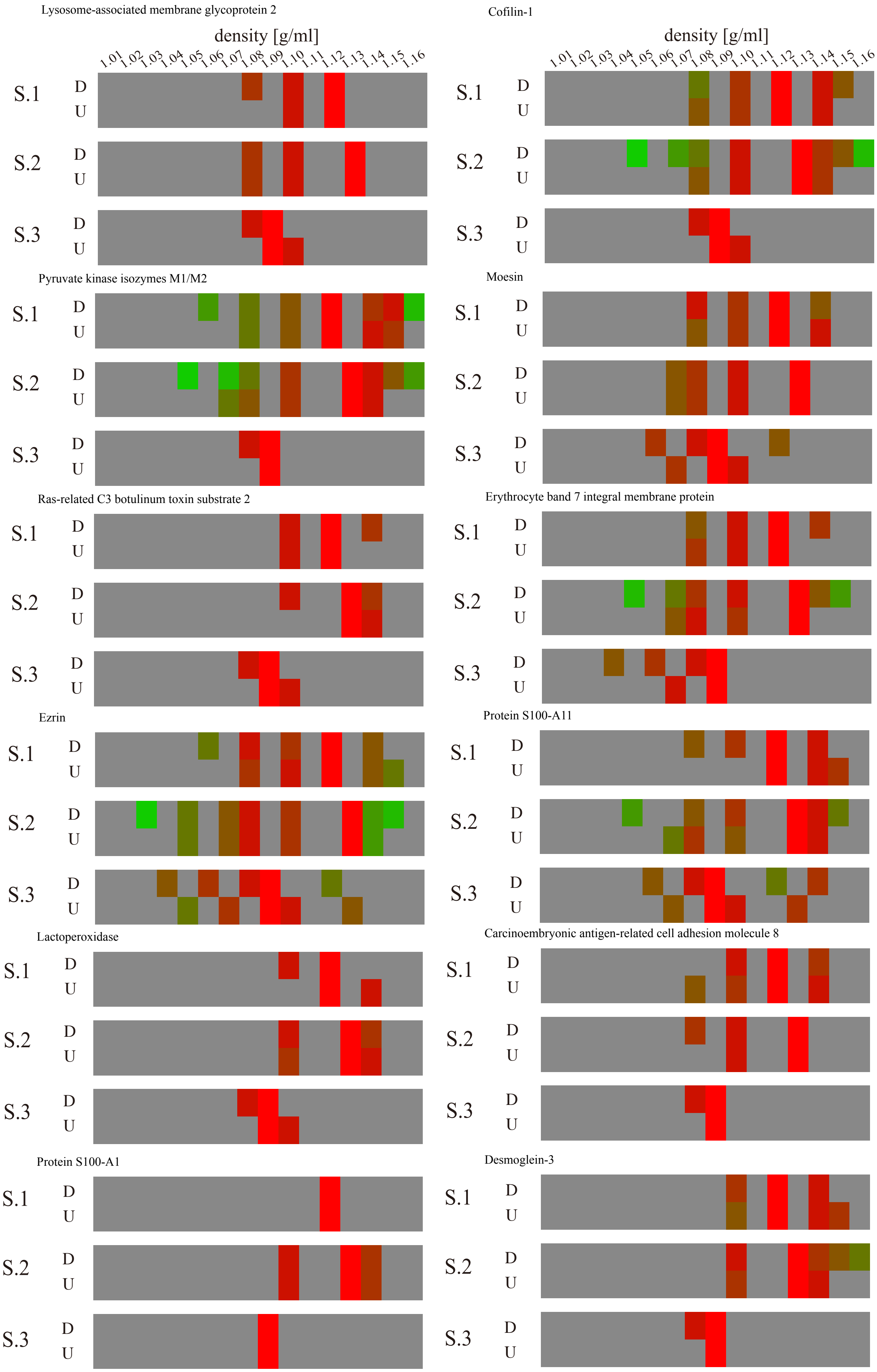
Lysosome-associated membrane glycoprotein 2
Cofilin-1
density [g/ml]
1.01 1.02 1.03 1.04 1.05 1.06 1.07 1.08 1.09 1.10 1.11 1.12 1.13 1.14 1.15 1.16
S.1
S.2
S.3
D
U
Pyruvate kinase isozymes M1/M2
Moesin
Ras-related C3 botulinum toxin substrate 2
Erythrocyte band 7 integral membrane protein
Ezrin
Protein S100-A11
Lactoperoxidase
Carcinoembryonic antigen-related cell adhesion molecule 8
Protein S100-A1
Desmoglein-3

IA-2

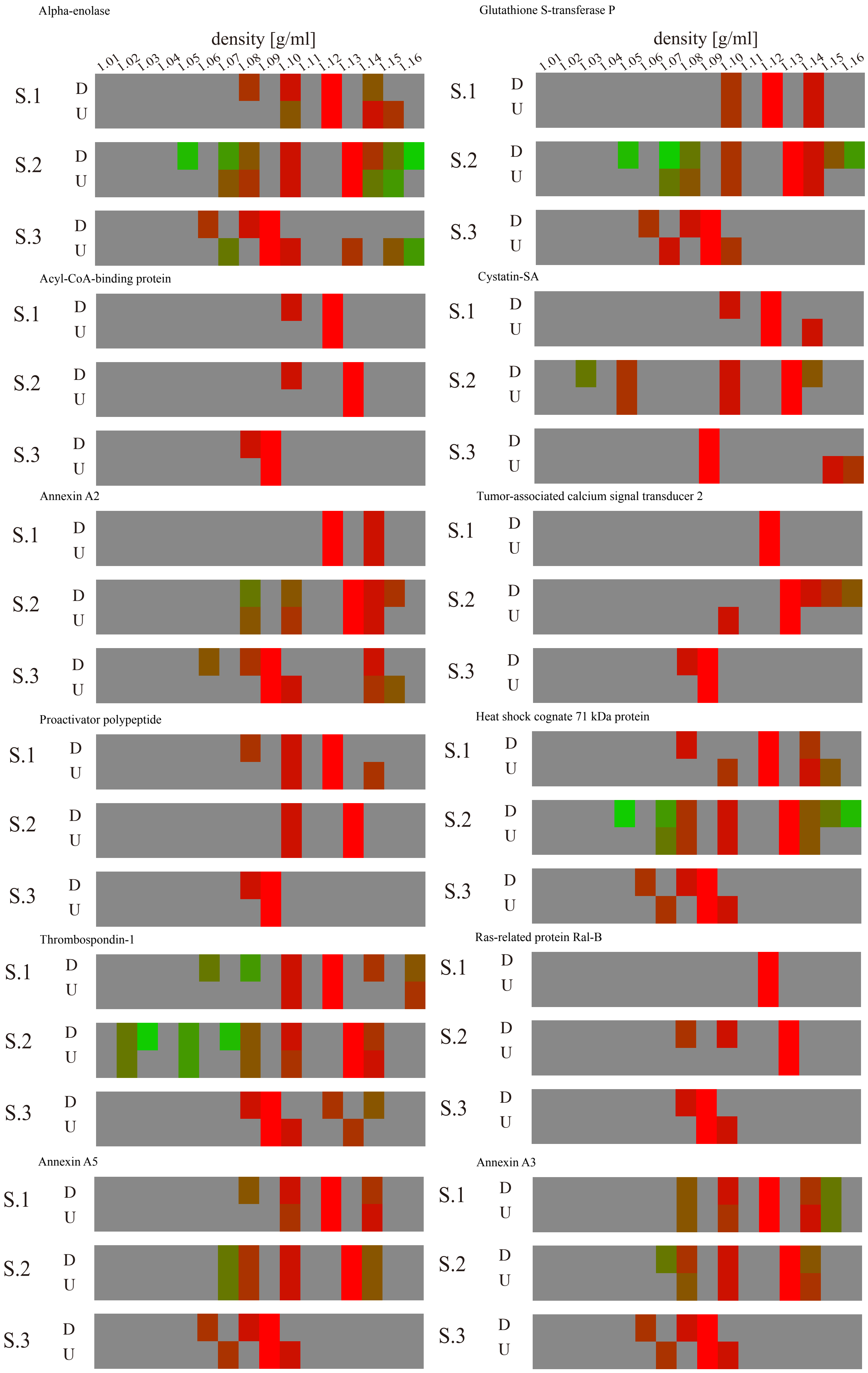
Alpha-enolase
density [g/ml]
1.01 1.02 1.03 1.04 1.05 1.06 1.07 1.08 1.09 1.10 1.11 1.12 1.13 1.14 1.15 1.16
S.1 D U
S.2 D U
S.3 D U
Acyl-CoA-binding protein
S.1 D U
S.2 D U
S.3 D U
Annexin A2
S.1 D U
S.2 D U
S.3 D U
Proactivator polypeptide
S.1 D U
S.2 D U
S.3 D U
Thrombospondin-1
S.1 D U
S.2 D U
S.3 D U
Annexin A5
S.1 D U
S.2 D U
S.3 D U
Glutathione S-transferase P
density [g/ml]
1.01 1.02 1.03 1.04 1.05 1.06 1.07 1.08 1.09 1.10 1.11 1.12 1.13 1.14 1.15 1.16
S.1 D U
S.2 D U
S.3 D U
Cystatin-SA
S.1 D U
S.2 D U
S.3 D U
Tumor-associated calcium signal transducer 2
S.1 D U
S.2 D U
S.3 D U
Heat shock cognate 71 kDa protein
S.1 D U
S.2 D U
S.3 D U
Ras-related protein Ral-B
S.1 D U
S.2 D U
S.3 D U
Annexin A3
S.1 D U
S.2 D U
S.3 D U

IA-2

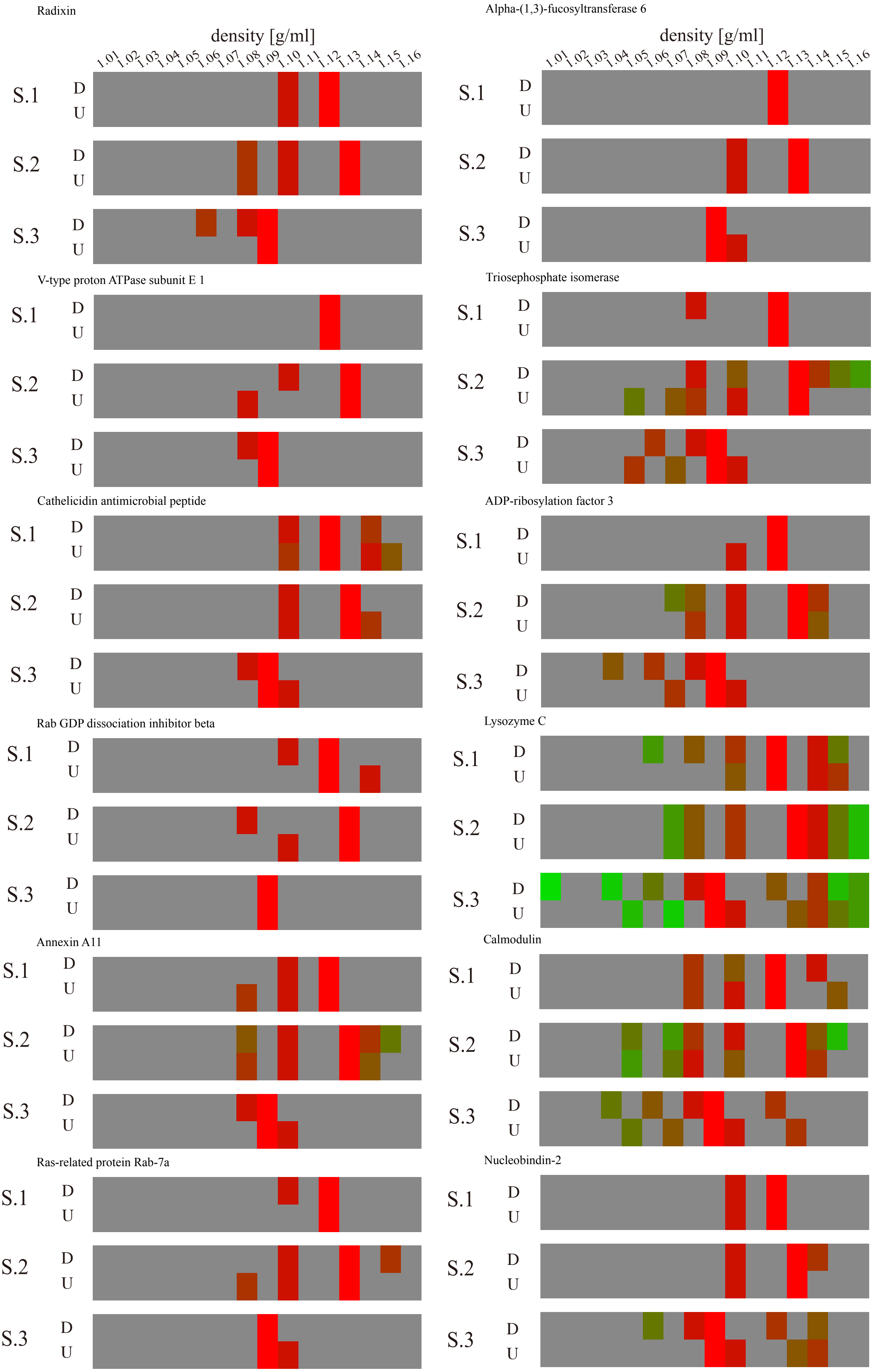
Radixin
density [g/ml]
1.01 1.02 1.03 1.04 1.05 1.06 1.07 1.08 1.09 1.10 1.11 1.12 1.13 1.14 1.15 1.16
S.1
S.2
S.3
D
U
V-type proton ATPase subunit E 1
Cathelicidin antimicrobial peptide
Rab GDP dissociation inhibitor beta
Annexin A11
Ras-related protein Rab-7a
Alpha-(1,3)-fucosyltransferase 6
Triosephosphate isomerase
ADP-ribosylation factor 3
Lysozyme C
Calmodulin
Nucleobindin-2

IA-2

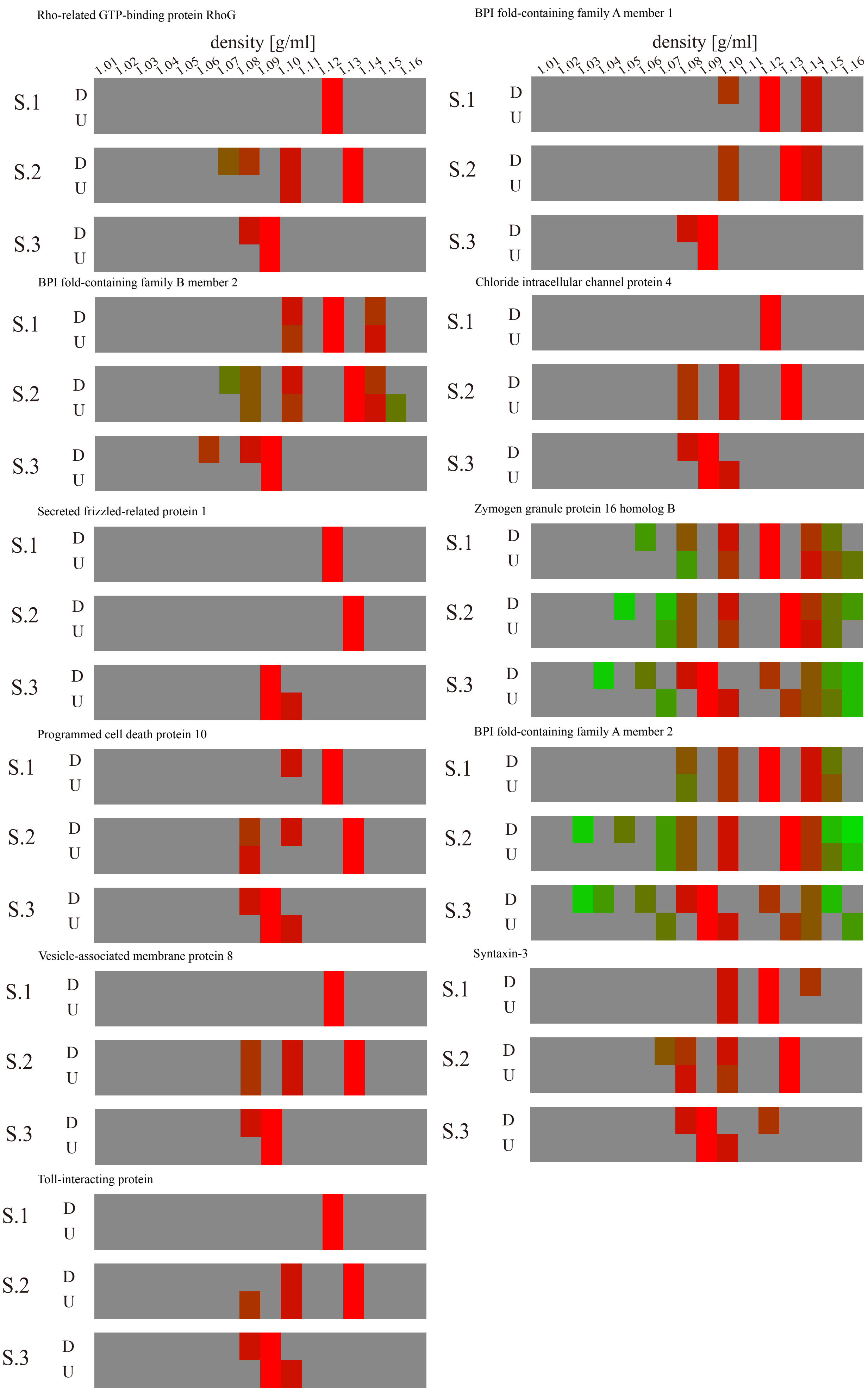
Rho-related GTP-binding protein RhoG
BPI fold-containing family A member 1
density [g/ml]
1.01 1.02 1.03 1.04 1.05 1.06 1.07 1.08 1.09 1.10 1.11 1.12 1.13 1.14 1.15 1.16
S.1
S.2
S.3
D
U
BPI fold-containing family B member 2
Chloride intracellular channel protein 4
Secreted frizzled-related protein 1
Zymogen granule protein 16 homolog B
Programmed cell death protein 10
BPI fold-containing family A member 2
Vesicle-associated membrane protein 8
Syntaxin-3
Toll-interacting protein
